# Neurons and molecules involved in noxious light sensation in *Caenorhabditis elegans*

**DOI:** 10.1093/g3journal/jkaf086

**Published:** 2025-04-16

**Authors:** Eva Dunkel, Ichiro Aoki, Amelie Bergs, Alexander Gottschalk

**Affiliations:** Buchmann Institute of Molecular Life Sciences, Department 14 (Biochemistry, Chemistry, Pharmacy), Goethe University, Max-von-Laue-Strasse 15, D-60438 Frankfurt, Germany; Institute for Biophysical Chemistry, Department 14 (Biochemistry, Chemistry, Pharmacy), Goethe University, Max-von-Laue-Strasse 9, Frankfurt D-60438, Germany; Buchmann Institute of Molecular Life Sciences, Department 14 (Biochemistry, Chemistry, Pharmacy), Goethe University, Max-von-Laue-Strasse 15, D-60438 Frankfurt, Germany; Institute for Biophysical Chemistry, Department 14 (Biochemistry, Chemistry, Pharmacy), Goethe University, Max-von-Laue-Strasse 9, Frankfurt D-60438, Germany; Buchmann Institute of Molecular Life Sciences, Department 14 (Biochemistry, Chemistry, Pharmacy), Goethe University, Max-von-Laue-Strasse 15, D-60438 Frankfurt, Germany; Institute for Biophysical Chemistry, Department 14 (Biochemistry, Chemistry, Pharmacy), Goethe University, Max-von-Laue-Strasse 9, Frankfurt D-60438, Germany; Buchmann Institute of Molecular Life Sciences, Department 14 (Biochemistry, Chemistry, Pharmacy), Goethe University, Max-von-Laue-Strasse 15, D-60438 Frankfurt, Germany; Institute for Biophysical Chemistry, Department 14 (Biochemistry, Chemistry, Pharmacy), Goethe University, Max-von-Laue-Strasse 9, Frankfurt D-60438, Germany

**Keywords:** LITE-1, negative phototaxis, neuropeptide, behavioral analysis, Ca^2+^ imaging, optogenetics, WormBase

## Abstract

Ultraviolet (UV) light is dangerous to unpigmented organisms, inducing photodamage of cells and DNA. The transparent nematode *Caenorhabditis elegans* detects light and exhibits negative phototaxis in order to evade sunlight. UV absorption is detected by the photosensor protein LITE-1 that also responds to reactive oxygen species. We investigated which neurons express LITE-1 and act as noxious photosensors and how they transmit this sensation to the nervous system to evoke escape behavior. We identified the interneuron AVG as a main focus of LITE-1 function in mediating the noxious light-evoked escape behavior, with minor roles of the interneuron PVT, the sensory ASK neurons, and touch receptor neurons. AVG is activated by blue light, and also its optogenetic stimulation causes escape behavior, while its optogenetic inhibition reduced escape. Signaling from AVG involves chemical neurotransmission, likely directly to premotor interneurons, and to other cells, by extrasynaptic signaling through the neuropeptide NLP-10. NLP-10 signaling is somewhat required for the acute response, yet is more important for maintaining responsiveness to repeated noxious stimuli. The source of NLP-10 in this context is largely AVG, yet also other cells contribute, possibly ASK. We uncover entry points of sensory information to neuronal circuits mediating noxious UV/blue light responses.

## Graphical Abstract

**Graphical Abstract:**
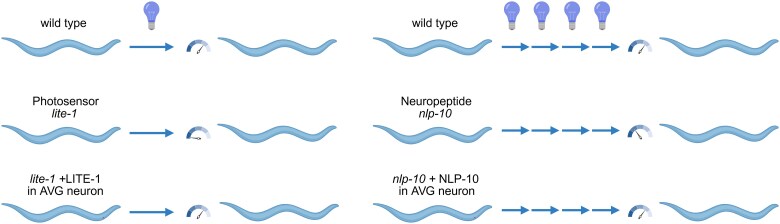

## Introduction

In the animal kingdom, different strategies exist to sense light and to adapt behavior accordingly. The eyes of vertebrates contain millions of photoreceptors, allowing them to sense their surrounding with great precision, which is essential for navigation and decision-making. The signals received by the retina are transmitted to the primary visual cortex via the lateral geniculate nucleus and processed in 2 streams in the cortex, eventually triggering behavioral output (Ungerleider 1982; Goodale and Milner 1992). Insects and arthropods have a comparatively simpler visual system, with thousands of sensory cells and complex downstream neural circuits that allow them to respond quickly, such as adapting their flight behavior to their environment (Land 1999). Least complex forms of animal vision result from photosensitive neurons and small circuits that can evoke tactic behaviors, for example, to avoid damage from ultraviolet (UV) light. UV-induced DNA damage involves formation of cyclobutane pyrimidine dimers, caused by absorption of UV-B photons (280–315 nm), and is linked to skin cancer development (Taylor 1994; Heil *et al*. 2011; Cadet and Douki 2018). The transparent nematode *Caenorhabditis elegans*, lacking organs for visual sensation, lives in soil or rotting biomass, which protects the animal from sunlight. Should it leave this habitat, negative phototaxis to UV and blue light, regulated by the LITE-1 protein, ensures its return to protected areas (Edwards *et al*. 2008).

The mechanism of UV light detection by LITE-1 is not yet clarified (Xiang *et al*. 2010; Bhatla and Horvitz 2015; Gong *et al*. 2016). LITE-1 [along with GUR-3, expressed in pharyngeal neurons and affecting light-evoked pharynx inhibition (Bhatla and Horvitz 2015)] is a member of a protein family including insect gustatory/olfactory receptors, which are ion channels (Saina *et al*. 2015; Butterwick *et al*. 2018; Del Marmol *et al*. 2021). Based on these similarities and on AlphaFold multimer modeling, LITE-1 was suggested to be a nociceptive light-activated ion channel (Evans *et al*. 2022; Hanson *et al*. 2023; Abramson *et al*. 2024). Moreover, LITE-1 is responsible for locomotion escape behavior, that is, either forward acceleration or a reversal, depending on whether the noxious light hits the posterior or anterior part of the animal, respectively (Edwards *et al*. 2008). This implies that both anterior and posterior cells mediate the escape response. Animals lacking LITE-1 show a largely decreased escape response. In preceding studies, a general rescue was obtained by expressing LITE-1 under its own promoter or specifically in AVG using the pF59E11.7 promoter in *lite-1(ce314)* mutants (Edwards *et al*. 2008; Aoki *et al*. 2024). mRNA of LITE-1 was detected in 15 different cells or cell types, being most abundantly expressed in the AVG neuron, followed by PVT and ASK neurons (Taylor *et al*. 2021) (Supplementary Fig. 1a and Table 1). These 3 neurons have been previously discussed as possible sites of action for LITE-1, or as photosensitive neurons (Edwards *et al*. 2008; Ward *et al*. 2008; Liu *et al*. 2010; Setty *et al*. 2022). AVG is a cholinergic interneuron, acting as a pioneer neuron for the right track of the ventral nerve cord (VNC) (Riddle *et al*. 1997; Pereira *et al*. 2015). Ablation of AVG in embryos leads to aberrant anatomical distribution of the axons of several ventral cord inter- and motor neuron, causing them to extend on the left side of the VNC (Durbin 1987; Garriga *et al*. 1993). The PVT neuron, exhibiting the second highest LITE-1 mRNA levels (Taylor *et al*. 2021), is a single interneuron located at the posterior end of the right VNC (Taylor *et al*. 2021) and is similarly important for normal VNC development (Ren *et al*. 1999). Removal of PVT in L1 stage animals results in positional changes of axons that were already embedded in the VNC (Aurelio *et al*. 2002). ASK is a head sensory neuron involved in chemotaxis behavior (Bargmann and Horvitz 1991). A previous study showed that a combination of sensory neurons (ASH, ASI, AWC, ADL, and mainly ASK, ASJ, and AWB) mediated avoidance of UVA light when LITE-1 was present (Liu *et al*. 2010). Other LITE-1-expressing cells include touch sensory neurons ALM and PLM.

It is unknown how the nociceptive signal perceived by LITE-1-expressing neurons is relayed to the remainder of the nervous system. Cells that receive innervation by these neurons are candidates for transforming the photoresponse into locomotion behavior (Supplementary Fig. 1b). These include AVA and AVB neurons, downstream of AVG, which are premotor interneurons that instruct reverse and forward locomotion, respectively. Also, the so-called first layer interneurons of the chemosensory navigation circuit, e.g. AIB and AIA neurons, which are innervated by PVT and ASK, relay signals to premotor interneurons (Gray *et al*. 2005). PVT and the touch receptor neurons also innervate AVK and DVA interneurons that affect navigation (Oranth *et al*. 2018; Aoki *et al*. 2024). However, LITE-1 signaling may also mediate a systemic signal that could alert many cells and tissues of danger by UV light. This idea is supported by several findings: (1) LITE-1 activation was shown to suppress the near-paralyzed phenotype of *unc-31* mutants (encoding the Ca^2+^ activator protein for secretion—CAPS), which largely lack the release of neuropeptides (Rupnik *et al*. 2000; Speese *et al*. 2007; Edwards *et al*. 2008; Steuer Costa *et al*. 2017; Yu *et al*. 2021). (2) *lite-1* mutants show abnormal locomotive behavior, such as reduced speed and impaired swimming (Edwards *et al*. 2008). (3) The absence of LITE-1 facilitates effects of the activation of a photosensitive cholinergic agonist (Damijonaitis *et al*. 2015). Thus, it appears plausible that LITE-1 may be responsible for the release of signaling molecules that generally affect locomotion. The signaling network of neuropeptides influences the whole organism (Smith *et al*. 2019; Taylor *et al*. 2021; Beets *et al*. 2023; Randi *et al*. 2023; Ripoll-Sanchez *et al*. 2023). One of the neuropeptides affecting *C. elegans* locomotion is NLP-10, which is expressed in multiple neurons, including AVG and PVT (Taylor *et al*. 2021). NLP-10 and its receptor, NPR-35, are required for the efficient response to noxious blue light, particularly to sustain the response upon repeated stimulation (Aoki *et al*. 2024).

Here, we aimed to identify the site of action for LITE-1 by cell-specific rescue. Our results suggest that AVG is the main site of action for the LITE-1-induced escape behavior in response to blue light exposure. The escape behavior was restored to wild-type level when rescuing LITE-1 in AVG, while suppressing neurotransmitter release from AVG resulted in impaired escape behavior. AVG Ca^2+^ levels are increased upon blue light illumination of the neurite, thus underlining the intrinsic reactivity of this neuron, whereas this reaction was abolished in *lite-1* mutants. We further identified AVG as a source of NLP-10 in this context, as specific expression of NLP-10 in AVG partially repressed habituation to repetitive blue light stimulation. However, effective suppression of habituation required additional neurons to express LITE-1, i.e. ASK and maybe PVT, the latter of which also expresses NLP-10. LITE-1-expressing neurons thus affect locomotion likely via synaptic networks and retain nociceptive sensitivity via the NLP-10 neuropeptide.

## Materials and methods

### Molecular biology

The constructs generated and/or used in this work can be found in Table 1.

**Table 1.** All constructs used and/or generated for this study.

Construct	Vector	Insert	Method	Source
pED068: *inx-18int2::GFP*	pIA446: *inx-18int2::lite-1::SL2::GFP* digested with ApaI–NheI	pIA210*: pflp-1::flp-1::GFP* digested with ApaI–NheI	T4 ligation	This study
pED070: *lim6-int3s::GFP*	pIA448: *lim6-int3s::lite-1::SL2::GFP* digested with ApaI–NheI	pIA210: *pflp-1::flp-1::GFP* digested with ApaI–NheI	T4 ligation	This study
pED071: *pmec-4::NLS::mScarlet*	pIA276: *pmec-4::NLS::GFP* digested with AgeI–EcoRI	pED072:: *pmec-4::mScarlet* amplified with primers oED118 5′-AAAAGAAACGCAAAGTACCGGTCATGGACAGTACAGAGGCAGTGATC-3′ and oED119 5′-ACCGGCGCTCAGTTGGAATTcTTAAGATCCTCCAGATCCTCC-3′	HiFi DNA assembly	This study
pED073: *inx-18int2::nlp-10::mScarlet*	pIA446: *inx-18int2::lite-1::SL2::GFP* digested with ApaI–MscI	pIA433: *pmec-4::nlp-10::mScarlet* digested with ApaI–MscI	T4 ligation	This study
pED080: *inx-18int2::Chrimson::P2A::GFP*	pIA273: *ptwk-47::Chrimson::P2A::GFP* digested with HindIII–XbaI	pIA446: *inx-18int2::lite-1::SL2::GFP* amplified with primers oMS018 5′-GGATACGCTAACAACTTGGA-3′ and oED126 5′-GATCAGCTCAGCCATGctagctgaaaatgttctatgttatgttag-3′	HiFi DNA assembly	This study
pED086: *inx-18int2::jRCaMP1b*	pED068: *inx-18int2::GFP* digested with NheI–ApaI	pXY09: *punc-17::jRCaMP1b* digested with NheI–ApaI	T4 ligation	This study
pED095: *inx-18int2::SNG-1::CRY2(535)*	pIA446: *inx-18int2::lite-1::SL2::GFP* digested with ApaI–NheI	pDV06: *punc-17::SNG-1::CRY2(535)* digested with ApaI–NheI	T4 ligation	This study
pIA423: *pZC190.6::lite-1::SL2::GFP*	pIA416: *pmyo-3::lite-1::SL2::GFP* digested with PciI–BamHI	pIA413: *pZC190.6::lite-1::GFP* digested with PciI–BamHI	T4 ligation	This study
pIA424: *pmec-4::lite-1::SL2::GFP*	pIA416: *pmyo-3::lite-1::SL2::GFP* digested with PciI–BamHI	pIA414: *pmec-4::lite-1::GFP* digested with PciI–BamHI	T4 ligation	This study
pIA446: *inx-18int2::lite-1::SL2::GFP*	pIA416: *pmyo-3::lite-1::SL2::GFP* digested with PciI - BamHI	pIA416: *pmyo-3::lite-1::SL2::GFP* amplified with oIA1596 5′-GCTGGCCTTTTGCTCACATG-3′ and oIA1607 5′-ctgcagaaGCTTATTTCATTTCCAAGTTG-3′ and genomic DNA amplified with oIA1608 5′-aatGAAATAAGCttctgcagttcgtaggtttttttcggg-3′ and oIA1609 5′-gctaatagaaaaaaaataagtaggtaacatgaaaaaaaatttttttg-3′ and genomic DNA amplified with oIA1610 5′-gttacctacttattttttttctattagcgtcatagatattttc-3′ and oIA1609 5′-TTGGCCAATCCCGGGGATCCGActgaaaatagtatcgatttttttg-3′	HiFi DNA assembly	This study
pIA448: *lim6-int3s::lite-1::SL2::GFP*	pIA416: *pmyo-3::lite-1::SL2::GFP* digested with PciI–BamHI	pIA416: *pmyo-3::lite-1::SL2::GFP* amplified with oIA1596 5′-GCTGGCCTTTTGCTCACATG-3′ and oIA1607 5′-ctgcagaaGCTTATTTCATTTCCAAGTTG-3′ and genomic DNA amplified with oIA1612 5′-aatGAAATAAGCttctgcagccttggtgcagag-3′ and oIA1609 5′-TTGGCCAATCCCGGGGATCcgtgaattttctaagcttcttgc-3′	HiFi DNA assembly	This study
pIA435: *pnlp-56::nlp-10::mScarlet*	pIA343: *pflr-1::nlp-10::mScarlet* digested with PstI and XbaI	Genomic DNA amplified with oIA1603 5′-TGAAATAAGCTTGGGCTGCAgTTTAAACGAACACACATACTTTTGG-3′ and oIA1604 5′-gAGCGATGTACCACATTCTAGACTGGAAGAGTTGAATCATATGG-3′	HiFi DNA assembly	This study
pIA338: *pttx-3::mScarlet*				Aoki *et al*. 2024

### *C. elegans* strains

*C. elegans* were cultivated on nematode growth medium (NGM) plates seeded with *E. coli* OP-50 bacteria (Brenner 1974). Unless described otherwise, histone-miniSOG animals (CZ20310 strain carrying *juSi164[pmex-5::his-72::miniSOG + Cbr-unc-119(+)] unc-119(ed3))* were labeled as WT. Strains used in this study are listed in Table 2.

**Table 2.** All strains used/generated in this study.

Strain	Genotype	Source
N2	*C. elegans* wild-type isolate	CGC
CB307	*unc-47(e307)*	CGC
*CZ20310*	*juSi164[pmex-5::his-72::miniSOG + Cbr-unc-119(+)] unc-119(ed3)*	CGC
ZX3711	*juSi164; lite-1(ce314)*	Aoki *et al*. (2024)
ZX3615	*nlp-10(zx29) juSi164*	Aoki *et al*. (2024)
ZX3712	*nlp-10(zx29) juSi164; lite-1(ce314)*	This study
ZX3763	*juSi164; lite-1(ce314); zxEx1500[pZC190.6::lite-1::SL2::GFP; pttx-3::mScarlet]*	This study
ZX3934	*juSi164; lite-1(ce314); zxIs269[inx-18-int2::lite-1::SL2::GFP; pttx-3::mScarlet]*	This study
ZX3935	*juSi164; lite-1(ce314); zxEx1544[lim-6-int3s::lite-1::SL2::GFP; pttx-3::mScarlet] #1*	This study
ZX3936	*juSi164; lite-1(ce314); zxEx1545[pmec-4::lite-1::SL2::GFP; pttx-3::mScarlet] #1*	This study
ZX3969	*juSi164; zxIs286[inx-18int2::Chrimson::P2A::GFP; pmec-4::mScarlet] #2*	This study
ZX3970	*juSi164; zxIs287[inx-18int2::RCaMP; pttx-3::GFP]*	This study
ZX3971	*juSi164; lite-1(ce314); zxIs287[inx-18-int2::RCaMP; pttx-3::GFP]*	This study
ZX3972	*nlp-10(zx29) juSi164; zxEx1547[inx-18-int2::nlp-10::mScarlet]*	This study
ZX3980	*nlp-10(zx29) juSi164; lite-1(ce314); zxEx155[inx-18-int2::nlp-10::mScarlet; inx-18int2::lite-1::P2A::GFP]*	This study
ZX3981	*juSi164; unc-47(e307); zxIs286[inx-18-int2::Chrimson::P2A::GFP; pmec-4::mScarlet]*	This study
ZX3995	*juSi164; zxEx1548[inx-18-int2::GFP; pttx-3::mScarlet]*	This study
ZX3996	*juSi164; zxEx1549[lim-6-int3s::GFP; pttx-3::mScarlet]*	This study
ZX3997	*juSi164; zxIs294[inx-18int2::SNG-1::CRY2(535); pmec-4::mScarlet]*	This study
ZX3998	*juSi164; lite-1(ce314); zxEx1550[inx-18-int2::lite-1::SL2::GFP; lim-6-int2::lite-1::SL2::GFP; pZC190.6::lite-1::SL2::GFP]*	This study
ZX3999	*juSi164; lite-1(ce314); zxIs286[inx-18-int2::Chrimson::P2A::GFP; pmec-4::mScarlet] #2*	This study
ZX4000	*nlp-10(zx29); juSi164; zxIs286[inx-18-int2::Chrimson::P2A::GFP; pmec-4::mScarlet] #2*	This study
ZX4054	*nlp-10(zx29); juSi164; lite-1(ce314); zxEx1567[inx-18-int2::NLP-10::mScarlet; pmec-4::NLS::GFP]*	This study
ZX4055	*juSi164; lite-1(ce314); zxIs269[pinx-18-int2::lite-1::SL2::GFP; pttx-3::mScarlet]; zxEx1568[lim6-int3s::LITE-1::SL2::GFP; pmyo-2::mCherry]*	This study
ZX4056	*juSi164; lite-1(ce314); zxEx1569[inx-18-int2::lite-1::SL2::GFP; pZC190.6::lite-1::SL2::GFP; pmec-4::NLS::mScarlet]*	This study
ZX4057	*nlp-10 (zx29); juSi164; zxEx1570[pnlp-56::NLP-10::mScarlet]*	This study

For the generation of transgenic lines, plasmid DNA in varying concentrations was injected into the gonads of adult hermaphrodites (Mello *et al*. 1991). Integration of plasmid DNA into the genome was achieved via activation of histone-miniSOG with blue light illumination 6 h after injection or after establishing an extrachromosomal strain (Noma and Jin 2018). Outcrossed *zxIs270* animals were shown to have identical behavior. An Arduino Duemilanove (Arduino, Turin, Italy) sent 3 Hz pulses to Power-LED-Module MinoStar (2.37 W, 36 lm, 30°, Signal Construct GmbH, Niefern-Oeschelbronn, Germany), which produced light at an intensity of 1.64 mW/mm^2^ for 36 min. All constructs were prepared using either T4 DNA Ligation (New England Biolabs, Ipswich, MA, USA) or HiFi DNA assembly (New England Biolabs, Ipswich, MA, USA). Primer sequences for *inx-18int2* and *lim-6int3 s* [for specific expression in AVG and in PVT, respectively (Oren-Suissa *et al*. 2016; Chien *et al*. 2017)] were adjusted to fit our cloning strategy.

### Behavioral assays

Twenty to forty L4 animals were picked on NGM plates 16 h prior to experiments (performed on the following day), unless stated otherwise. Plates were kept at 22°C in the dark. Crawling behavior of animals was recorded with the Multi-Worm-Tracker (MWT), as previously described (Swierczek *et al*. 2011; Aoki *et al*. 2024). For the measurements investigating the response to blue light, an LED ring illuminated the plate with 1.0 mW/mm^2^ at 470 nm. Where appropriate, activation of Chrimson was performed with an intensity of 0.3 mW/mm^2^ at 620 nm wavelength. After the initial data processing by the Choreography software, which evaluates the speed and body bending of the animals, the data were analyzed and visualized with the help of custom MATLAB scripts (Aoki *et al*. 2024). Baseline speed was normalized to 30 s before (first) blue light illumination. For each measurement (indicated as *N*), the mean speed of recorded animals (indicated as a range for the number of animals *n*) was used for further analysis. All figures presenting crawling speed show mean values ± standard error (SEM). Box or violin plots represent the median of the means (*N* measurements) with 25/75% quartiles. Means for each measurement were plotted as individual scatters.

Representative videos of strains were recorded with a LabVIEW-based custom software MS-Acqu under the same conditions as measurements were done. Displaying recording time and indication of the illumination period and the compression of the original videos were performed with Clipchamp (Microsoft).

### Fluorescence imaging

Animals were placed on pads composed of 2.5% agarose dissolved in M9 buffer and immobilized with levamisole-hydrochloride (20 mM; Sigma-Aldrich, St. Louis, Missouri, USA) and 0.1 µm PolyBeads (Polysciences Inc., Warrington, Pennsylvania, USA). Images were obtained with a Kinetix22 camera (Teledyne Photometrics, Tucson, Arizona, USA), mounted on a Zeiss AxioObserver Z1 with a 40 × oil immersion objective (EC Plan-NEOFLUAR 40x/1.3 Oil DIC. 420462-9900. Carl Zeiss, Oberkochen, Germany). Fluorescent proteins were excited with 460 or 590 nm Prior Lumen LEDs (Lumen 100, Prior Scientific, Cambridge, UK), which were regulated by µManager 2.0. An AOTFcontroller script (https://github.com/micro-manager/mmCoreAndDevices/blob/main/DeviceAdapters/Arduino/AOTFcontroller/AOTFcontroller.ino) was applied to control Z-stack recordings with an Arduino Uno.

To obtain representative images of fluorescent signal, an EGFP/mCherry double band pass filter (450–490 nm and 555–590 nm excitation, 520/630 nm beamsplitter and 520/20; 630/30 nm emission; AHF Analysentechnik, Germany) was used. For the verification of LITE-1::SL2::GFP expression in ASK, animals were incubated in DiI staining solution (Invitrogen, Thermo Fisher Scientific Inc., Darmstadt, Germany). A 1 mM stock solution was diluted 1:100 in M9 buffer, and young adult animals were transferred into the staining solution and rotated for 4 h in the dark. Before imaging, animals were washed twice in M9 to remove dye residues. Schematic representations of whole animals with the position of AVG, ASK, or PVT were created in BioRender.

### Calcium imaging assays

Animals expressing AVG::jRCaMP1b were prepared in the same manner as for the fluorescence imaging and observed under a Zeiss AxioObserver Z1 with a 100 × oil immersion objective (EC Plan-NEOFLUAR 100x/1.3 Oil DIC. 420490-9900) and equipped with an RCaMP filter set (479/585 excitation, 605 nm beamsplitter, and 647/57 nm emission, AHF Analysentechnik, Germany). Two hundred millisecond light pulses (2 Hz) at 590 nm were used to excite the jRCaMP1b signal, while additional 200 ms light pulses (2 Hz) at 460 nm (0.34 mW/mm²) were applied between 30 and 40 s of recordings, to evoke the noxious light response. A custom script running on Arduino created an illumination pattern, to solely obtain images during yellow light illumination. For quantitative analysis, a ROI was placed along the axon of AVG, while a second ROI was placed on the body wall of animals, near the AVG axon. Signal intensity was measured with Fiji (ImageJ 1.53c, National Institutes of Health, USA). Afterwards, the intensity values obtained from the second ROI were subtracted from those of the first ROI, as background correction. Baseline values were calculated 10 s before the start of the illumination with blue light, and the signal intensity is presented as follows:


$$\frac{△F}{F_{0}}=\frac{F-F_{0}}{F_{0}}$$

For soma-specific stimulation, an LCD projector was used (Hitachi cp-x605, modified according to a previous publication) (Stirman *et al*. 2012). To relay the projected image to the focal plane of the sample, a Zeiss Axiovert 200 microscope was modified by removing the lenses of the epifluorescence optical train and inserting an accessory tube lens (165 mm) in the optical path between projector and objective according to the mentioned study (Stirman *et al*. 2012). Additionally, the microscope was equipped with a 40 × oil immersion objective (EC Plan-NEOFLUAR 40x/1.3 Oil DIC. 420462-9900. Carl Zeiss, Oberkochen, Germany) and an EMCCD camera (Evolve 512 Delta, Photometrics, Tucson, Arizona, USA). Projector calibration, hardware synchronization, and projection of preselected colored ROIs within a defined pulse protocol were performed using custom-written code for µManager (Edelstein *et al*. 2010) (http://micro-manager.org), as Beanshell scripts (Supplementary Code 1 “Projector_calibration.bsh” and Supplementary Code 2 “Pulse_protocol_patterned_stimulation.bsh”, provided as supplementary files). RCaMP fluorescence was continuously excited using an HBO lamp equipped with a 580 nm filter (580/23 nm excitation, AHF Analysentechnik, Germany) at 180 µW/mm^2^. After AVG soma and neurite were recorded for 10 s, the soma was illuminated additionally with blue light by the projector for 10 s (200 µW/mm^2^, 80R/20T beam splitter) (F21-002, AHF Analysentechnik, Germany, RCaMP filter set), followed by 10 s of recordings to determine any offset responses.

### Coelomocyte imaging

Coelomocytes of *nlp-10* or *nlp-10; lite-1* mutants expressing AVG::NLP-10::mScarlet were imaged under the Zeiss AxioObserver Z1 with a 40 × oil immersion objective (EC Plan-NEOFLUAR 40x/1.3 Oil DIC. 420,462–9900. Carl Zeiss, Oberkochen, Germany), equipped with the identical RCaMP filter as in calcium imaging assays. NLP-10::mScarlet was excited using an LED (590 nm, Lumen 100, Prior Scientific, Cambridge, UK). To obtain differences in the fluorescence signal ratio of anterior coelomocytes and the AVG soma, signal intensities were measured in animals without blue light illumination and in animals that underwent the same illumination pattern as in behavioral assays (4 × 30 s 1.0 mW/mm^2^ illumination at 470 nm, with 270 s dark intervals). Animals were recorded immediately after the repetitive stimulation was performed. Values obtained by a third ROI, set onto the body wall near the AVG soma, were subtracted from the intensity values obtained from coelomocytes and from the soma, as a background correction.

### Statistical analysis

Statistical analysis, as well as presentation of obtained data in graphs, were done using Prism 9.4.1 (GraphPad software). For comparisons of more than 2 data sets, 1- or 2-way ANOVAs were applied with Tukey as post hoc analysis. For comparisons of 2 normally distributed data sets, an unpaired *t*-test was applied. For comparison of data sets that were not normally distributed, a Mann–Whitney test was applied. Asterisks indicate the significance levels of *P* < 0.05 (*), *P* < 0.01 (**), *P* < 0.001 (***), and *P* < 0.0001 (****).

## Results

### The interneuron AVG is the main site of action of LITE-1

To identify possible sites of action for LITE-1, we wanted to express the protein in *lite-1(ce314)* mutants under different promoters that are specific or even exclusive for neurons that natively express the *lite-1* gene. These cells include, based on single-cell RNAseq data and by abundance of the *lite-1* mRNA (Taylor *et al*. 2021), the neurons AVG, PVT, and ASK, as well as the touch receptor neurons (TRNs) ALM and PLM (Supplementary Fig. 1a and Table 1). Lower abundance was reported for ASG, PHA, PHB, RIF, RMD, and a number of pharyngeal neurons. We restricted our analysis to the cells with most abundant *lite-1* expression, AVG, PVT, ASK, and the TRNs. Some of these cells make synaptic connections to cells of the motor and navigation system (Supplementary Fig. 1b). The following promoters were cloned and assessed for their expression as transcriptional GFP fusions (Supplementary Fig. 2): The second intron of the *inx-18* gene (*inx-18-int2*) (Oren-Suissa *et al*. 2016) instructed expression of GFP in a single neuron with the cell body in the retrovesicular ganglion and a process reaching all along the VNC toward the tail (Supplementary Fig. 2a). The expression matched the reported morphology of AVG (White *et al*. 1986). A part of the third intron of the *lim-6* gene (*lim-6-int3 s)* (Chien *et al*. 2017) drove expression in a neuron with a cell body in the preanal ganglion and extended a process through the VNC and around the nerve ring, where it formed several varicosities (Supplementary Fig. 2b). This morphology is in line with details reported for the PVT neuron (White *et al*. 1986; Witvliet *et al*. 2021), and localization relative to a marker for the AIY neuron corresponded with this interpretation. For the ASKL/R neurons, we tested several promoters based on scRNAseq data (Taylor *et al*. 2021). We settled for the *ZC190.6* promoter, which expressed in 2 neurons with cell bodies in the lateral nerve ring ganglia, and processes to the amphid sensory organs (Supplementary Fig. 2c). Also, these cells stained positive for the lipophilic dye DiI, which can enter the sensory endings of these neurons. Yet, 2 additional, unknown neurons, one in the tail and one about halfway between the pharyngeal terminal bulb and the vulva (possibly, ALM or AVM neurons, based on position and morphology), were expressing this promoter. For the TRNs, we used the well-established *mec-4* promoter (Chalfie and Sulston 1981).

**Fig. 1.**
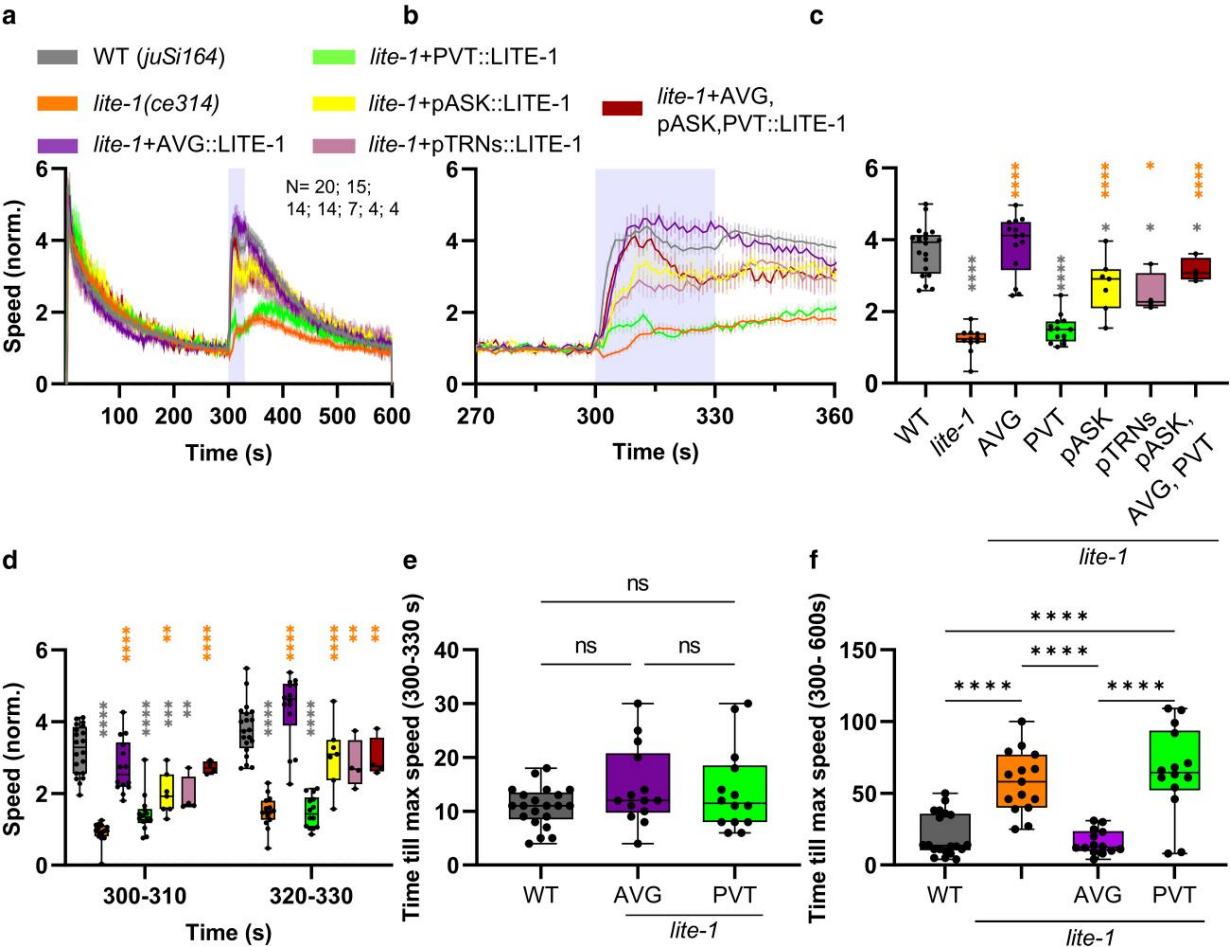
LITE-1 is required mainly in AVG and in ASK and TRNs as secondary site of action for noxious light-evoked escape behavior. a) Mean crawling speed (±SEM) of animals before (0–299 s), during (300–330 s), and after (331–600 s) blue light illumination. Tested strains were wild type, *lite-1(ce314)*, and *lite-1* mutants expressing LITE-1 solely in neurons AVG, PVT, ASK, and in TRNs, or simultaneously in AVG, PVT, and ASK. Speed was normalized to the mean crawling speed from 270 to 299 s. Illumination is indicated by the shaded regions behind the graphs. *N* = 20, 15, 14, 14, 7, 4, and 4 experiments, with *n* = 20–32 animals each, respectively. b) Close-up of 270–360 s, including the illumination period. c) Comparison of speed among the indicated strains during the illumination period. Median with 25/75 quartiles; whiskers indicate minimum and maximum values. One-way ANOVA with Tukey test (*P < 0.05; ****P < 0.0001). Gray asterisks indicate significant difference to wild type, others as indicated. Note that in a comparison of the time windows of 305–320 s, the triple rescue strain was not different to wild type. Full statistical analysis can be found in Supplementary Data File 1. d) Comparison of speeds between strains during the first and last 10 s of illumination. Median with 25/75 quartiles; whiskers indicate minimum and maximum values. Two-way ANOVA with Tukey test. Gray and orange asterisks: Significant differences to wild type and *lite-1*, respectively (*P < 0.05; **P < 0.01; ***P < 0.001; ****P < 0.0001). e) Time until maximum speed is reached during illumination period. Median with 25/75 quartiles; whiskers indicate minimum and maximum values. One-way ANOVA with Tukey test (ns, not significant). f) Time until maximum speed is reached after stimulus onset. Median with 25/75 quartiles; whiskers indicate minimum and maximum values. One-way ANOVA with Tukey test (****P < 0.0001).

We then assessed transgenic animals expressing LITE-1 cDNA from these promoters for their potential to rescue the blue light-evoked escape response. As controls, we used wild-type animals (or rather, the *juSi164* background, used for transgene integration into the genome; *juSi164* expresses the miniature single oxygen generator (miniSOG) fused to histone HIS-72 (Noma and Jin 2018)). Importantly, *juSi164* animals show locomotion and blue light-evoked escape behavior that is indistinguishable from wild-type N2 animals (Supplementary Fig. 3). For some experiments, we also used *juSi164; lite-1(ce314)* mutants. During the blue light stimulation (1 mW/mm²), *lite-1* animals showed an increase in speed (1.2-fold) that was significantly lower than that of wild-type animals, which increased their speed about 4-fold (Fig. 1, a–c; note that data were normalized to the respective mean during the 270–299 s just preceding the light stimulus). This difference was not only detectable in statistical analysis but also upon visual examination of the animals’ behavior (Supplementary Videos 1 and 2). *lite-1* mutants expressing LITE-1 in AVG showed a clear increase in speed, up to 3.9-fold, i.e. a significant, complete rescue of the light-evoked escape behavior. For animals expressing LITE-1 in the PVT neuron, there was only a minor initial speed increase upon noxious blue light. Thus, PVT contributes only to a minor extent to the light-evoked escape behavior. Strains expressing LITE-1 in AVG presented a slightly delayed onset of the reaction; however, the maximum speed during the illumination period was not reached significantly later than in wild-type controls (Fig. 1, d and e). The time to reach the maximum speed during the illumination period for animals carrying PVT::LITE-1 likewise did not differ from wild-type or AVG::LITE-1 animals. However, PVT::LITE-1 animals exhibited a pronounced, clearly delayed offset response following the end of the light stimulus by about 40 s (Fig. 1f). Analysis of the reversal rate of animals before, during, and after the illumination period in 10 s bins showed increased reversal behavior of *lite-1* animals, indicating some residual light responsiveness, possibly through GUR-3 (Bhatla and Horvitz 2015), but otherwise did not reveal clear differences between rescued animals and wild type (Supplementary Fig. 4, a–c).

Next, we tested the ASK neurons and the TRNs, including PLM and ALM, as possible sites of action for LITE-1. Both rescue strains showed a speed increase upon blue light stimulation that was statistically significantly larger than for *lite-1* controls, yet not as pronounced as for wild-type animals (Fig. 1, a–d). Last, we tested a strain expressing all 3 rescue constructs, i.e. for AVG, PVT, and ASK. The combined expression of LITE-1 in neurons AVG, PVT, and ASK led to escape behavior that was almost identical in its amplitude and speed development as in wild-type animals, resulting in a clear rescue of the *lite-1* phenotype (Fig. 1, a–d). However, the AVG single neuron rescue was somewhat more similar to wild type. We note that the triple rescue was not a chromosomally integrated transgene, in contrast to the single neuron rescue; depending on the time window of analysis, i.e. 305–320 s, the triple rescue was not significantly different to wild type. Given the extent of rescue, AVG seems to be a main focus of activity of LITE-1.

### AVG responds to blue light, depending on LITE-1, and is required for the behavioral response to blue light

**Fig. 2.**
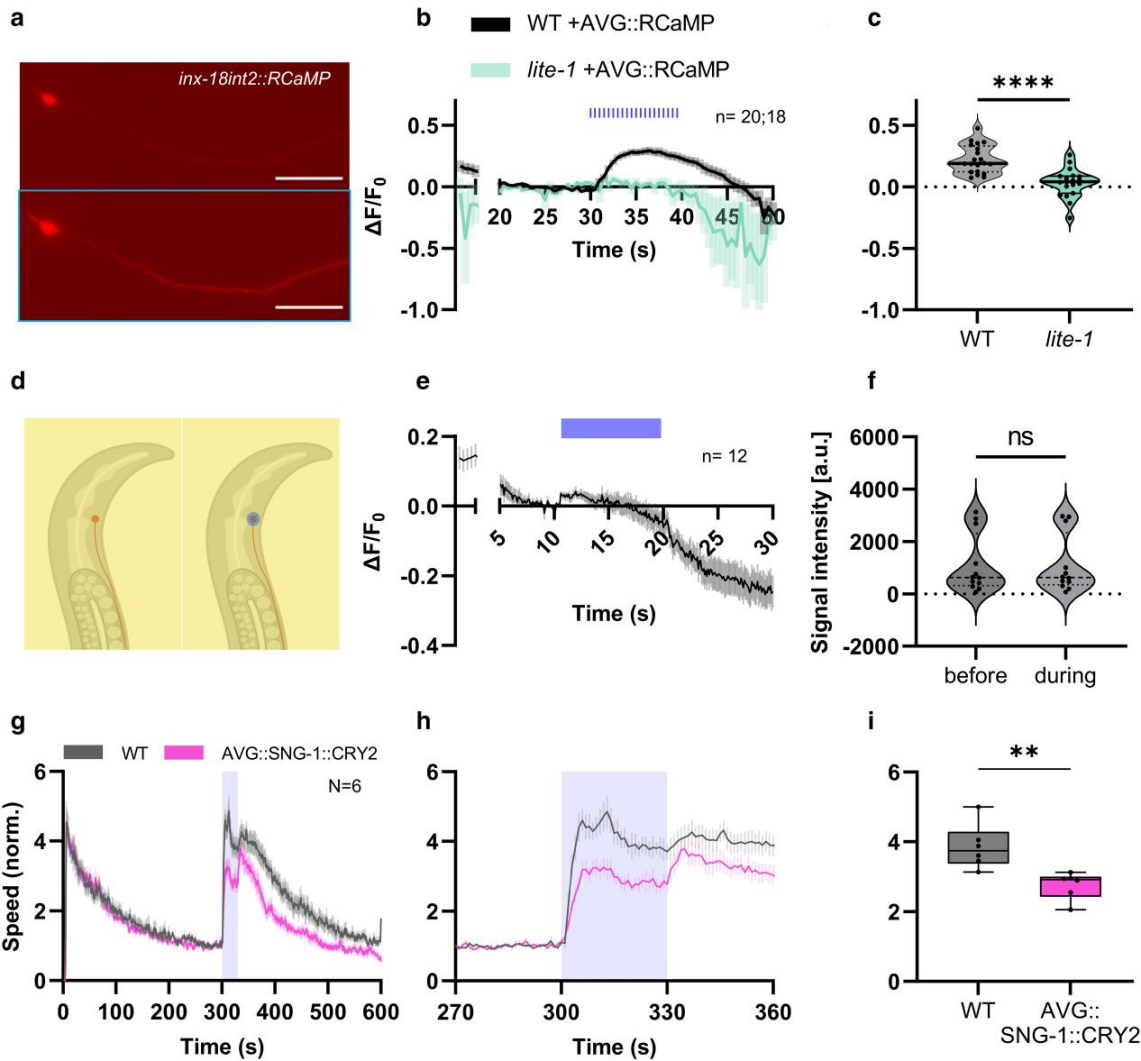
The AVG neuron is activated by blue light and releases a signal by chemical neurotransmission. a) Exemplary images of RCaMP signal in AVG (cell body and axon, anterior is left) before (top) and right after (bottom, outlined box) blue light application. Scale bars: 50 µm. b) Mean (±SEM) change of fluorescence intensity of RCaMP in AVG before (20–29 s), during (30–39 s), and after (40–50 s) application of blue light pulses (indicated by bars). *n* = 20 animals for wild type and *n* = 19 for *lite-1*. *F*_0_ was calculated during seconds 20–29. c) Mean RCaMP signal intensity during blue light application of individual animals. Median (thick line) and 25/75 quartiles (dotted or thin lines). Unpaired *t*-test (****P < 0.0001). d) Schematic illustration of illumination of the AVG soma (right) with blue light, restricted to the soma. AVG soma (filled circle) was placed in the center of the field of view. Animals were exposed to yellow light continuously (shaded box). Blue light was solely presented to the soma (blue), using a video projector. BioRender license LB281CGZH2. e) Mean (±SEM) change of fluorescence intensity of RCaMP in AVG before (5–9 s), during (10–19 s), and after (20–30 s) application of blue light (indicated by blue bar); *n* = 12. *F*_0_ was calculated during seconds 5–9. f) Mean RCaMP signal intensity of the AVG process in individual animals, before and during blue light application. Median (thick line) and 25/75 quartiles (dotted or thin lines). Paired *t*-test (ns, not significant). g) Mean crawling speed (±SEM) of animals before (0–299 s), during (300–330 s), and after (331–600 s) blue light illumination. Tested strains were wild type and animals expressing optoSynC (optogenetic tool for immobilization of synaptic vesicles) in AVG. Speed was normalized to the mean crawling speed from 270 to 299 s. Illumination indicated by blue shade. *N* = 6 experiments with 27–38 animals each. h) Close-up of locomotion speed 30 s before, during, and after illumination. i) Comparison of crawling speed during illumination. Median with 25/75 quartiles; whiskers indicate minimum and maximum values. Unpaired, 2-tailed *t*-test (*P* = 0.0052).

To verify that endogenous LITE-1 function can activate AVG, we examined the effect of blue light on AVG Ca^2+^ levels. The red fluorescent Ca^2+^ indicator RCaMP, which can be combined with blue light stimulation (Akerboom *et al*. 2013), showed a significant increase in signal intensity in both process and soma, when the animals were illuminated with blue light (Fig. 2, a–c; Supplementary Fig. 5, a and b). This demonstrated that blue light causes an activation of AVG, leading to a rise of the Ca^2+^ concentration. In *lite-1* mutants, the RCaMP signal increase was absent (Fig. 2, b and c; Supplementary Fig. 5, a and b). Thus, AVG gets activated by blue light, and this effect depends on LITE-1. A negative control expressing a calcium-insensitive fluorescent protein did not change in its signal intensity (Supplementary Fig. 5c). The question arises, where in AVG the LITE-1 protein may be localized. Illuminating solely the soma of AVG with blue light led only to a minimal increase in fluorescence signal of RCaMP in neurites, which was not significantly different before and during stimulation (Fig. 2, d–f). These findings suggest that illumination of the whole cell, including the neurite, activates AVG; light stimulation restricted to the soma is not sufficient to increase Ca^2+^ levels. This indicates that LITE-1 protein may be localized to the neuronal process, and not the soma of AVG, in line with a sensory function that would cover the body and not only the anterior end of the animals, and in line with a previous report showing LITE-1 protein expression in the tail process of the AVG neuron (Edwards *et al*. 2008).

To further analyze how AVG propagates the light-induced signal to downstream cells affecting locomotion, we targeted chemical synaptic transmission. We inhibited the mobility of synaptic vesicles (SVs) and thus their recruitment for transmitter release at the active zone plasma membrane, specifically in AVG, using the *opto*genetic *syn*aptic vesicle *c*lustering tool optoSynC (Vettkötter *et al*. 2022). This protein consists of the SV protein SNG-1 and CRY2olig, a light-dependent oligomerizing variant of cryptochrome 2. We previously showed that pan-neuronal expression of this tool suppressed the reaction to intense blue light to a level similar as in *lite-1* mutant background (Vettkötter *et al*. 2022). By expressing optoSynC specifically in AVG and activating it by blue light, we could reduce neurotransmitter release from AVG. This led to a significantly decreased escape behavior, yet did not abolish it (Fig. 2, g–i). Potentially, in addition to normal AVG synaptic output *via* acetylcholine, optoSynC may have additionally targeted transmission by neuropeptides from dense core vesicles, provided SNG-1 is part of these vesicles, too.

### Depolarization of AVG leads to increased locomotion speed, which is affected in the absence of GABA

**Fig. 3.**
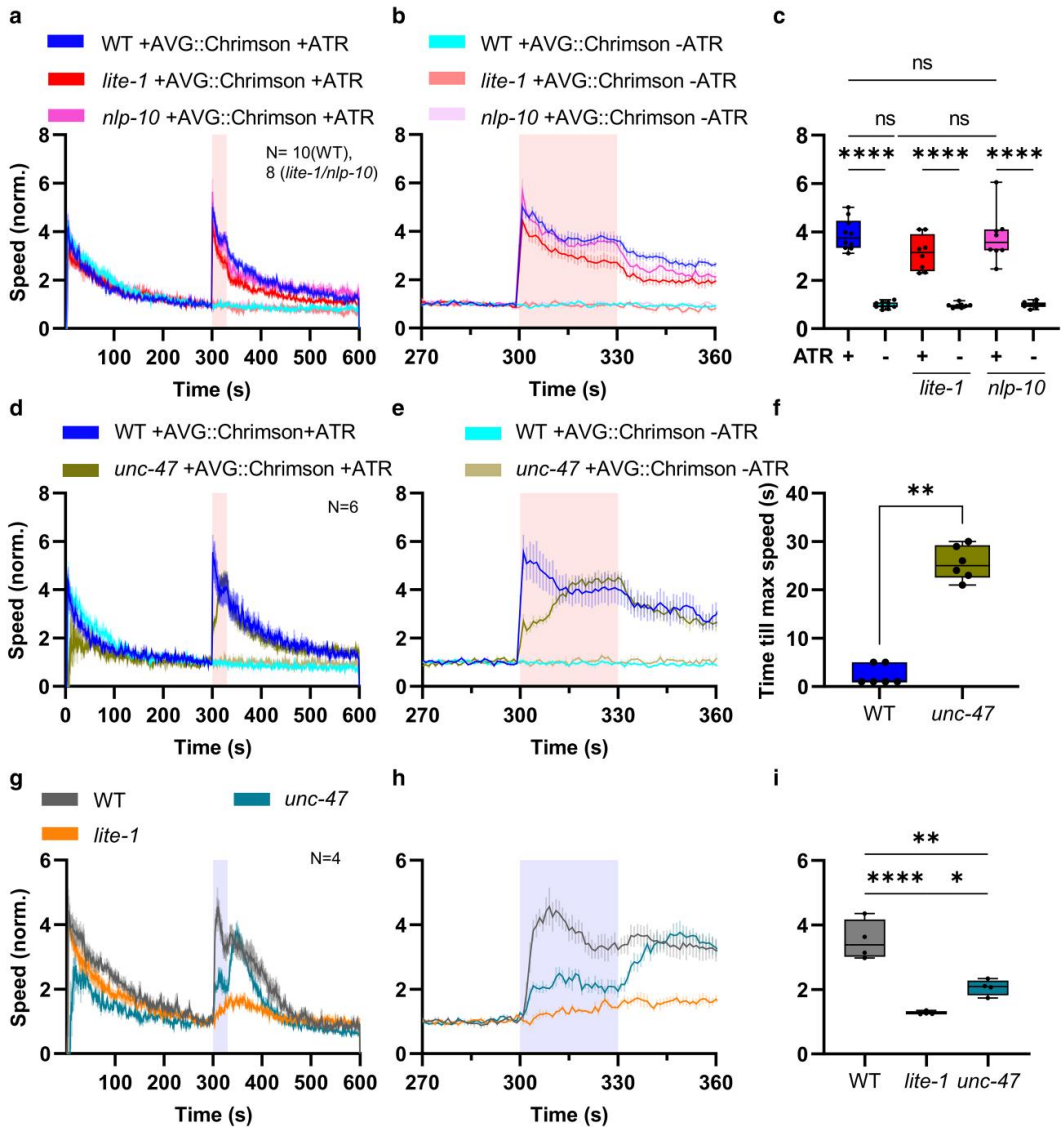
Optogenetic stimulation of AVG triggers escape behavior. a) Mean crawling speed (±SEM) of animals expressing Chrimson in AVG in wild type (*N* = 10), *lite-1(ce314)* (*N* = 8), and *nlp-10(zx29)* (*N* = 8 with 19–35 animals for each strain) was analyzed before, during, and after red light illumination (shaded regions behind the graphs). Crawling speeds were normalized to the respective mean crawling speeds from 270 to 299 s. Animals without exposure to ATR served as a negative controls. b) Close-up of locomotion speed 30 s before, during, and after illumination. c) Statistical analysis of data in a) and b), during red light illumination. Median with 25/75 quartiles; whiskers indicate minimum and maximum values. Two-way ANOVA with Tukey post hoc analysis (ns, not significant; ****P < 0.0001). d and e) As in a) and b), but for wild type or *unc-47(e307)* animals expressing AVG::Chrimson that were either exposed to ATR or not. *N* = 6 with 26–31 animals each. f) Time until animals reached their maximum speed during the illumination. Median with 25/75 quartiles; whiskers indicate minimum and maximum values. Mann–Whitney test (*P* = 0.0022). g) Mean crawling speed (±SEM) of wild type, *lite-1*, or *unc-47* animals before (0–299 s), during (300–330 s), and after (331–600 s) blue light illumination. Crawling speed was normalized to the mean crawling speed from 270 to 299 s. Illumination is indicated by the shaded regions behind the graphs. *N* = 4 experiments with 22–32 animals each. h) Close-up of locomotion speed 30 s before, during, and after illumination. i) Mean speed during the illumination period. Median with 25/75 quartiles; whiskers indicate minimum and maximum values. One-way ANOVA with Tukey test (*P < 0.05; **P < 0.01; ***P < 0.001).

LITE-1 was recently suggested to be a light-activated ion channel (Hanson *et al*. 2023). This implies that depolarization of AVG should cause the same behaviors as activation of LITE-1. At the same time, it would demonstrate that AVG-specific expression of the LITE-1 protein is inducing cellular signaling based on depolarization. We thus used the AVG promoter to drive expression of Chrimson, a red light-activated channelrhodopsin (ChR) variant (Klapoetke *et al*. 2014; Oda *et al*. 2018), specifically in AVG (Supplementary Fig. 6). When animals expressing AVG::Chrimson were fed the ChR chromophore all-*trans* retinal (ATR) and illuminated with red light (620 nm, 0.3 mW/mm²), we observed a clear increase in locomotion speed (Fig. 3, a and b; Supplementary Video 3). This effect was not observed in animals that were not treated with ATR beforehand, ruling out any behavioral effect on the animals caused by the red light illumination (Fig. 3c).

We compared the effect of AVG photodepolarization in *lite-1* and *nlp-10* mutants. NLP-10 was shown to contribute to the effects of blue light stimulation of animals, particularly to keep up a strong effect during repeated stimulation (Aoki *et al*. 2024). For both mutant backgrounds, AVG::Chrimson activation evoked a robust and immediate reaction of animals (Fig. 3, a–c). Even though *lite-1* mutants showed a slightly weaker reaction upon AVG activation, this difference was not statistically significant, and the responses were observed only in animals treated with ATR. This verifies that AVG depolarization evokes similar behaviors like activation of LITE-1 in AVG and that AVG is a major focus of function of LITE-1, in line with the hypothesis that LITE-1 is a depolarizing ion channel (Hanson *et al*. 2023). The nature of the transmitter that AVG uses to evoke the acute signal is as yet unclear.

AVG was reported to be cholinergic; however, a recent study revealed that AVG also expresses the vesicular GABA transporter (vGAT) UNC-47 (Wang *et al*. 2024). Thus, to assess if the (mostly) inhibitory neurotransmitter GABA might contribute to the AVG-evoked escape behavior, we crossed AVG::Chrimson into *unc-47(e307)* mutants. Upon red light illumination and therefore Chrimson activation, an increase in speed was still detectable (Fig. 3, d–f). Nevertheless, the behavior of *unc-47* animals differed from wild-type animals: The immediate reaction to the stimulation was weaker and animals slowly increased their speed level, reaching the peak much later than wild-type animals (Supplementary Fig. 7, a and b, and Video 4). These results suggest that GABA might facilitate the immediate response upon AVG activation, though it is not essential for it. Since our readout is based on locomotion, and GABA signaling is crucial for proper function of the neuromuscular junction (Bamber *et al*. 1999), we tested if *unc-47* mutants are generally affected for LITE-1-induced behavioral responses. *unc-47* animals had reduced basal locomotion speed (Supplementary Fig. 7, c and d), so we normalized the data to the time before the light stimulus was presented (Fig. 3, g and h). While wild-type animals showed the previously observed rapid and robust (ca. 4-fold) speed increase, this response was attenuated for *unc-47* mutants, which increased their speed only about 2-fold, though was significantly more than *lite-1* mutants (Fig. 3i). The *unc-47* mutant animals showed a much larger offset speed increase after the end of the blue light stimulus, for unknown reasons (Fig. 3, g and h). In sum, GABA signaling affects the acuteness of LITE-1 evoked escape responses, either directly or indirectly.

### AVG-derived NLP-10 rescues the behavioral habituation to repeated blue light stimulation

**Fig. 4.**
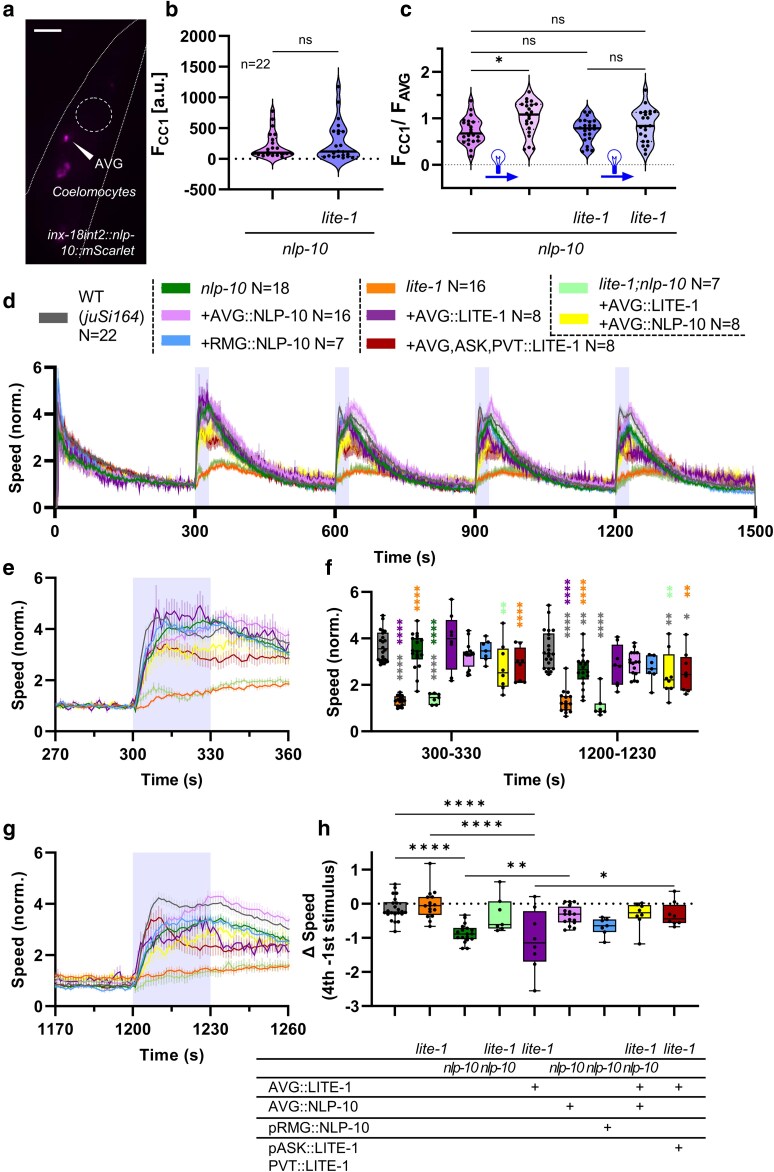
Expression of NLP-10 in AVG rescues habituation to repetitive blue light stimulation. a) Representative image of NLP-10::mScarlet fluorescence, expressed specifically in AVG. Arrowhead indicates AVG soma; additional signal was seen in coelomocytes. Scale bar indicates 50 µm. b) Fluorescence intensity of mScarlet in *nlp-10* and *nlp-10; lite-1* mutants in anterior coelomocytes (CC1). Unpaired *t*-test (ns, not significant). c) Fluorescence intensity of mScarlet in *nlp-10* and *nlp-10; lite-1* mutants with (second and fourth column) and without (first and third column) repetitive blue light illumination. One-way ANOVA with Tukey post hoc analysis (*P < 0.05; ns, not significant). d) Mean crawling speed (±SEM) of the indicated strains during repetitive blue light stimulation at 300–330, 600–630, 900–930, and 1200–1230 s, as indicated by shaded regions behind the graphs. Crawling speed was normalized to the mean crawling speed from 270 to 299 s. *N* experiments per strain, as indicated, with 23–41 animals each. e and g) Close-ups of locomotion speed 30 s before, during, and after the first and the fourth illumination period, respectively. f) Comparison of crawling speed during the first (300–330 s) and fourth illumination (1200–1230 s). Median with 25/75 quartiles; whiskers indicate minimum and maximum values. Two-way ANOVA with Tukey post hoc analysis was applied (*P < 0.05; **P < 0.01; ***P < 0.001; ****P < 0.0001). Color of asterisks indicates significant differences to the respective strain. Full statistical analyses can be found in Supplementary Data File 1. h) Differences of mean speeds of fourth illumination, deduced by speed levels at first illumination. Median with 25/75 quartiles; whiskers indicate minimum and maximum values. One-way ANOVA with Tukey test (*P < 0.05; **P < 0.01; ***P < 0.001; ****P < 0.0001).

As we showed recently, the NLP-10 neuropeptide suppresses the behavioral habituation to repetitive blue light exposure (Aoki *et al*. 2024). Since AVG (and PVT) also express NLP-10 (Supplementary Table 1 and Fig. 1a), it is possible that NLP-10, released from AVG upon noxious blue light stimulation, contributes to the sustained responsiveness to the noxious stimulus, as long as it persists. To examine the contribution of NLP-10, released from AVG, we expressed mScarlet, fused to the prepropeptide of NLP-10, in AVG. We verified the expression pattern and found that, as expected, mScarlet signal was visible in the AVG cell body. Signal was also observed in coelomocytes, which are scavenger cells that endocytose and filter the body fluid (Sieburth *et al*. 2007), thus demonstrating that NLP-10 is released from AVG (Fig. 4a). To investigate whether NLP-10 is released upon noxious blue light illumination, we measured the fluorescence intensity of mScarlet in the AVG soma and in coelomocytes. While the initial values in coelomocytes did not differ in *nlp-10* mutants and *nlp-10; lite-1* double mutants without illumination, the ratio of the fluorescence signal in coelomocytes over the signal in the AVG soma increased in animals that were exposed to noxious blue light (4 times 30 s over 15 min; Fig. 4, b and c). Since this was not observed in the double mutants, the release of NLP-10 over steady state levels from AVG appears to be LITE-1 dependent.

Next, we subjected animals to repeated blue light exposures and analyzed their locomotion behavior (Fig. 4, d–h). During the first illumination, *nlp-10(zx29)* mutants and *nlp-10* mutants expressing NLP-10 only in AVG showed responses similar to wild-type animals (Fig. 4, d–f); however, the *nlp-10* mutants (and, to some extent, the AVG-specific NLP-10 rescue animals) showed a smaller amplitude and a slower onset of the response. Thus, NLP-10 may be required for the acuteness of the blue light escape behavior, though the source of NLP-10 may not be exclusively AVG. As observed before (Aoki *et al*. 2024), the extent of speed increase became smaller for each consecutive stimulus in *nlp-10* mutants (Fig. 4, d–g), while it was maintained at the high level of wild-type animals also in the *nlp-10* animals expressing NLP-10 in AVG. To more directly analyze this, we subtracted the mean speed of the fourth stimulus from that of the first stimulus (Fig. 4h), showing that the AVG::NLP-10 animals performed significantly better than *nlp-10* mutants and were not different from wild type. Expression of NLP-10 in RMG, which expresses higher *nlp-10* mRNA levels in comparison to AVG (Supplementary Table 1) (Taylor *et al*. 2021), did not rescue the habituation effect in *nlp-10* mutants (Fig. 4, g and h), verifying that AVG is a relevant source of NLP-10 neuropeptides in this context. Overall, AVG-derived NLP-10 led to a rescue of enhanced habituation to noxious blue light-evoked escape behavior in *nlp-10* mutants. Given the time course of the speed increase during illumination, however, AVG is likely not the only site of action of NLP-10 regarding this phenotype (Aoki *et al*. 2024).

We further analyzed this effect in *lite-1; nlp-10* double mutants, as well as in cell-specific rescue strains for both genes. As expected, the double mutants showed only a minimal speed increase upon the first light stimulation, just as *lite-1* mutants (Fig. 4, d–g). The reduced responsiveness of the *lite-1* or the *lite-1; nlp-10* double mutants for the first stimulus was partially rescued by expressing LITE-1 or LITE-1 and NLP-10, respectively, in AVG. However, expressing both LITE-1 and NLP-10 in the double mutant reduced the overall response. Yet, they appeared to maintain their sensitivity to repetitive stimulation, as the habituation levels were similar to wild type: Comparing the habituation (fourth minus first stimulus; Fig. 4h) showed that while rescuing LITE-1 in AVG in *lite-1* mutants led to a robust rescue of the defect of the initial response, the speed difference between the fourth and first stimulation was significantly larger than in wild type, a similar effect as in *nlp-10* mutants. Therefore, we asked if other cells expressing LITE-1 and/or NLP-10 may be required for maintaining full responsiveness. We thus rescued LITE-1 in AVG, PVT, and ASK simultaneously. This maintained the nociceptive responsiveness upon the fourth stimulus significantly better than in *lite-1* mutants expressing LITE-1 only in AVG. Further measurements revealed that expression of LITE-1 in AVG and ASK simultaneously results in a wild type–like behavior for repetitive blue light stimulation, while animals expressing LITE-1 only in AVG or additionally in PVT still showed a reduction of sensitivity (Supplementary Fig. 8).

**Fig. 5.**
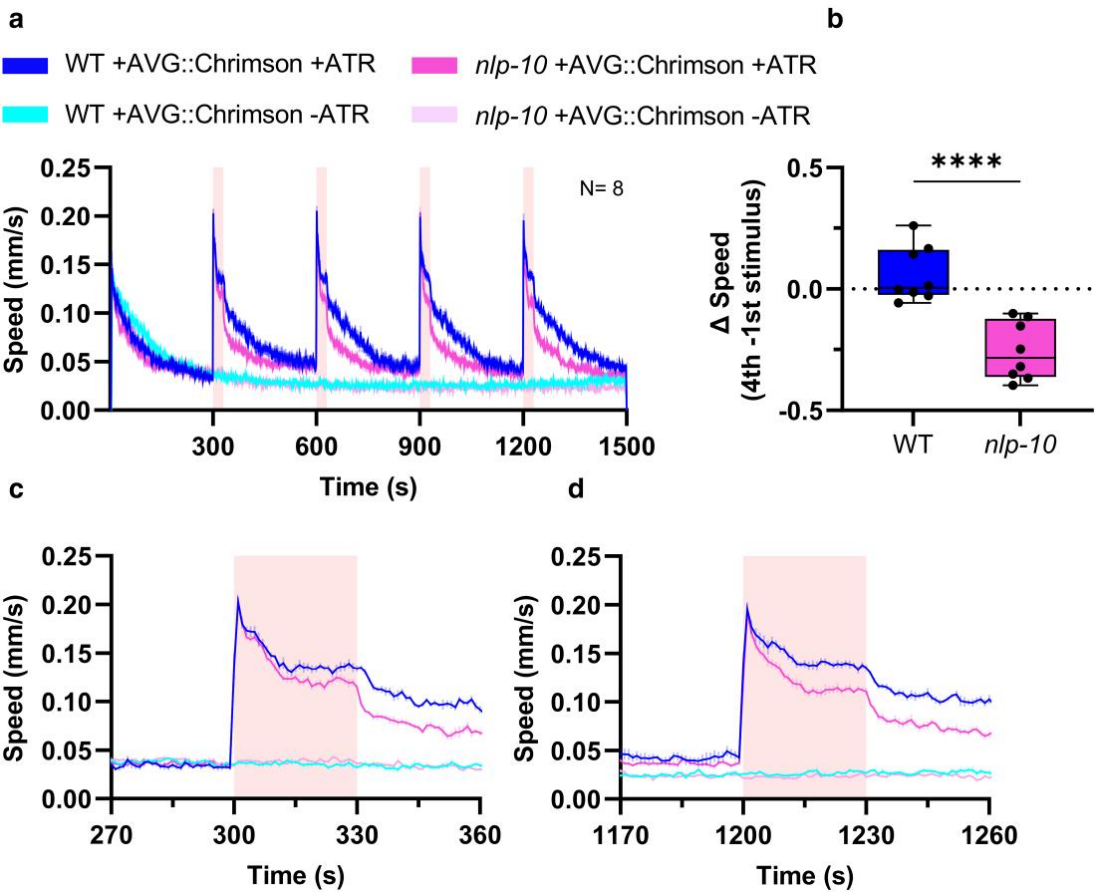
*nlp-10* mutants exhibit reduced sensitivity to repetitive optogenetic activation of AVG. a) Mean crawling speed (±SEM) of wild type and *nlp-10(zx29)* animals expressing Chrimson in AVG during repetitive red light stimulation at 300–330, 600–630, 900–930, and 1200–1230 s, as indicated by shaded regions behind the graphs. *N* = 8 experiments with 19 to 27 animals each. b) Difference of speed level upon first vs fourth illumination; mean speed during the fourth illumination was subtracted from mean speed during the first illumination. Median with 25/75 quartiles; whiskers indicate minimum and maximum values. Unpaired, 2-tailed *t*-test (****P < 0.01). c and d) Close-ups of locomotion speed 30 s before, during, and after the first or the fourth illumination period, respectively.

To probe if NLP-10 release is also required to maintain responsiveness to AVG photostimulation using AVG::Chrimson, we compared the behavioral responses in wild type and in *nlp-10* mutants (Fig. 5). AVG stimulation caused robust escape behavior following each of the four stimuli (Fig. 5, a–d). In *nlp-10* mutants, this was slightly, but significantly, affected. While *nlp-10* mutants behaved similarly during stimulus onset, their speed levels decreased more quickly during the illumination period, which resulted in a slower speed level during the fourth stimulation (Fig. 5, a and d). Comparing speed levels between the first and the fourth illumination, *nlp-10* mutants showed a stronger habituation than wild-type animals (Fig. 5b). In sum, blue light-evoked nociceptive behavior largely involves the stimulation of AVG and to some extent also ASK and PVT. Furthermore, AVG is a source of NLP-10, enabling to maintain nociceptive responsiveness. However, other cells that release NLP-10 are required to fully maintain the blue light (AVG)-evoked locomotion speed increase.

## Discussion

Here, we analyzed which neurons contribute to the UV-blue light avoidance of *C. elegans*. The neuron AVG, which expresses highest levels of LITE-1 mRNA, is also the most important neuron driving the escape behavior. Other cells, particularly ASK and touch receptor neurons, contribute to this response. PVT, the neuron expressing the second highest amount of *lite-1* mRNA, is not required for the acute response, but also contributes to the overall maintenance of the nociceptive responsiveness upon repeated exposure. This aspect of LITE-1-induced signaling requires the neuropeptide NLP-10, for which a major source in this context is again AVG. Which signal is the driver for the acute locomotion speed increase, however, remains unclear. It is likely to be sought in the neuronal circuits downstream of AVG, specifically the premotor interneurons for forward locomotion. However, AVG also forms synapses with backward premotor interneurons. It remains to be identified how the 2 signaling aspects may be coordinated. The fact that AVG appears to be both cholinergic and GABAergic could imply specific, opposing signal output to the respective interneuron type, promoting net forward acceleration. However, at stage, this is speculation.

Intense short-wave light causes damage in living organisms, which can even be fatal. Recent studies describe LITE-1 as an ion channel, which is light activated, and acts as a receptor for photons and H_2_O_2_-coincidence via PRDX-2 (Quintin *et al*. 2022; Hanson *et al*. 2023). Another member of the gustatory receptor family of odorant receptors of is GUR-3 (Bhatla and Horvitz 2015). The site of action of these proteins is partially known. Spatial resolution of the photosensation does not occur by a light-sensitive organ, but by the location of the neurons and/or their processes, potentially in varicosities, expressing LITE-1 and GUR-3 along the body. That is, GUR-3 is restricted to neurons of the pharyngeal nervous system and mediates a stop of pharyngeal pumping, along with LITE-1 (Bhatla and Horvitz 2015). As LITE-1 is only expressed in a handful of neurons, our goal was to identify its site of action in the context of the blue light-induced escape behavior.

Previous work implicated AVG, PVT, and ASK in the context of (LITE-1 mediated) negative phototaxis based on expression of a reporter construct and on cell ablation experiments (Edwards *et al*. 2008; Ward *et al*. 2008; Liu *et al*. 2010). When noxious light is applied on the posterior part of the animal, AVG might evoke escape behavior, whereas in the anterior end of the animal, PVT (but not AVG) extends into the nerve ring, suggesting that PVT may mediate LITE-1 mediated reversal behavior. ASK ablation (alongside other ciliated neurons such as ASJ, AWB and ASH that extend amphid sensory endings to the nose tip) resulted in a reduced reversal response, whereas LITE-1 rescue in ASK in *lite-1* mutants lifted the intensity of photophobic behavior to about 50% of wild-type animals (Ward *et al*. 2008; Liu *et al*. 2010). In our hands, the expression of LITE-1 exclusively in AVG rescued most of the missing photophobic acceleration response of *lite-1* mutants.

The cholinergic neuron AVG also expresses the vGAT UNC-47, which is involved in GABA and possibly glycine transport (McIntire *et al*. 1993; Eastman *et al*. 1999; Gendrel *et al*. 2016). Photostimulation *via* Chrimson in AVG resulted in an immediate speed increase in wild type, as well as in *lite-1* and *nlp-10* mutants; however, *unc-47* mutants responded with an initially reduced speed increase. Thus, GABA (or glycine) might play a role in the translation of AVG activation to locomotion output. The delay in time until the maximum speed was reached is not caused by the generally uncoordinated crawling behavior of these mutants, since exposing *unc-47* animals to blue light resulted in an immediate onset of the response as in wild type, though with reduced amplitude. Nevertheless, to rigorously demonstrate an involvement of GABA in AVG signaling will require analyzing animals with a cell-specific knockdown of *unc-47* mRNA or a cell-specific rescue in an *unc-47* mutant background. Silencing AVG chemical transmission by clustering of synaptic vesicles reduced the reactivity of the animals to noxious light. Previously, it was suggested that LITE-1 activation somehow stimulates cholinergic neurons (Edwards *et al*. 2008); however, it was unclear whether this was a direct or indirect effect, due to release of signaling molecules that act at the organismic level to activate cholinergic signaling. LITE-1 was found to suppress the activity of a blue light (470 nm) photoswitchable version of acetylcholine, AzoCholine, when animals are preexposed to UV light (350 nm), possibly due to a systemic signal released in response to LITE-1 activation (Damijonaitis *et al*. 2015). Interestingly, *unc-31* mutants that have an impaired release of neuropeptides and are almost paralyzed display a normal reaction to blue-violet light (Edwards *et al*. 2008); thus, the signal may not be a neuropeptide. Yet, given the AVG::optoSynC results, it should be released by vesicular exocytosis.

In sum, nociceptive signaling downstream from AVG might mostly involve cholinergic signaling, as well as NLP-10 neuropeptides to sustain the response. NLP-10 might have long-range effects (Ludwig and Leng 2006; van den Pol 2012). Our previous study suggests that NLP-10 is crucial to inhibit habituation to noxious blue light stimulation, which could be rescued by the expression of the neuropeptide in different neurons, including AVK (Aoki *et al*. 2024) that is innervated by PVT via chemical and electrical synapses. Also expression of NLP-10 in AVG rescued the compromised speed increase noticed in *nlp-10* mutants, indicating that AVG is a site of action for NLP-10. Interestingly, a similar effect as seen in *nlp-10* mutants was observed in animals expressing LITE-1 only in AVG. However, when LITE-1 was expressed in AVG and ASK, animals maintained their responsiveness. This implies that while AVG is the main contributor to the initial response to noxious light avoidance, ASK is crucial for keeping up sensitivity. It should be noted that other neurons express NLP-10 at high levels, e.g. RMG, but expression in RMG was not sufficient for preventing the habituation of the blue light response. Thus, it appears that NLP-10 must be released in a specific (local, or timed) manner, probably acting on the circuits mediating the respective, habituating behavior, which may be probed by cell-specific expression of the NLP-10 receptor, NPR-35 (Aoki *et al*. 2024).

## Supplementary Material

jkaf086_Supplementary_Data

## Data Availability

Data are mostly on-line measurements from the MWT, for which no video is saved. These data, all quantitative imaging data and the respective statistical analysis, are summarized in Supplementary Data File 1, sorted for each figure of the paper. Exemplary videos can be found as Supplementary Videos 1–4 in the supplementary files. All supplementary figures and table are provided in Supplementary File 1. Strains and plasmids are available upon request. Matlab scripts used to analyze MWT data can be found at Zenodo (https://doi.org/10.5281/zenodo.13958554). Code for blue light illumination of the soma while Ca^2+^ imaging are provided as Beanshell scripts in the Supplementary Code 1 “Projector_calibration.bsh” and Supplementary Code 2 “Pulse_protocol_patterned_stimulation.bsh”). Supplemental material available at G3 online.

## References

[jkaf086-B1] Abramson J, Adler J, Dunger J, Evans R, Green T, Pritzel A, Ronneberger O, Willmore L, Ballard AJ, Bambrick J, et al 2024. Accurate structure prediction of biomolecular interactions with AlphaFold 3. Nature. 630(8016):493–500. doi:10.1038/s41586-024-07487-w. 38718835 PMC11168924

[jkaf086-B2] Akerboom J, Carreras Calderón N, Tian L, Wabnig S, Prigge M, Tolö J, Gordus A, Orger MB, Severi KE, Macklin JJ, et al 2013. Genetically encoded calcium indicators for multi-color neural activity imaging and combination with optogenetics. Front Mol Neurosci. 6:2. doi:10.3389/fnmol.2013.00002. 23459413 PMC3586699

[jkaf086-B3] Aoki I, Golinelli L, Dunkel E, Bhat S, Bassam E, Beets I, Gottschalk A. 2024. Hierarchical regulation of functionally antagonistic neuropeptides expressed in a single neuron pair. Nat Commun. 15(1):9504. doi:10.1038/s41467-024-53899-7. 39489735 PMC11532408

[jkaf086-B4] Aurelio O, Hall DH, Hobert O. 2002. Immunoglobulin-domain proteins required for maintenance of ventral nerve cord organization. Science. 295(5555):686–690. doi:10.1126/science.1066642. 11809975

[jkaf086-B5] Bamber BA, Beg AA, Twyman RE, Jorgensen EM. 1999. The Caenorhabditis elegans unc-49 locus encodes multiple subunits of a heteromultimeric GABA receptor. J Neurosci. 19(13):5348–5359. doi:10.1523/JNEUROSCI.19-13-05348.1999. 10377345 PMC6782323

[jkaf086-B6] Bargmann CI, Horvitz HR. 1991. Chemosensory neurons with overlapping functions direct chemotaxis to multiple chemicals in C. elegans. Neuron. 7(5):729–742. doi:10.1016/0896-6273(91)90276-6. 1660283

[jkaf086-B7] Beets I, Zels S, Vandewyer E, Demeulemeester J, Caers J, Baytemur E, Courtney A, Golinelli L, Hasakioğulları İ, Schafer WR, et al 2023. System-wide mapping of peptide-GPCR interactions in C. elegans. Cell Rep. 42(9):113058. doi:10.1016/j.celrep.2023.113058. 37656621 PMC7615250

[jkaf086-B62] Brenner S . 1974 The genetics of Caenorhabditis elegans. Genetics. 77:71–94. 4366476 10.1093/genetics/77.1.71 PMC1213120

[jkaf086-B8] Bhatla N, Horvitz HR. 2015. Light and hydrogen peroxide inhibit C. elegans feeding through gustatory receptor orthologs and pharyngeal neurons. Neuron. 85(4):804–818. doi:10.1016/j.neuron.2014.12.061. 25640076 PMC4408612

[jkaf086-B9] Butterwick JA, Del Mármol J, Kim KH, Kahlson MA, Rogow JA, Walz T, Ruta V. 2018. Cryo-EM structure of the insect olfactory receptor Orco. Nature. 560(7719):447–452. doi:10.1038/s41586-018-0420-8. 30111839 PMC6129982

[jkaf086-B10] Cadet J, Douki T. 2018. Formation of UV-induced DNA damage contributing to skin cancer development. Photochem Photobiol Sci. 17(12):1816–1841. doi:10.1039/c7pp00395a. 29405222

[jkaf086-B11] Chalfie M, Sulston J. 1981. Developmental genetics of the mechanosensory neurons of Caenorhabditis elegans. Dev Biol. 82(2):358–370. doi:10.1016/0012-1606(81)90459-0. 7227647

[jkaf086-B12] Chien J, Devkota R, Yosef N, Mörck C. 2017. Regulation of axon guidance by the Wnt receptor Ror/CAM-1 in the PVT guidepost cell in Caenorhabditis elegans. Genetics. 207(4):1533–1545. doi:10.1534/genetics.117.300375. 28993416 PMC5714464

[jkaf086-B13] Damijonaitis A, Broichhagen J, Urushima T, Hüll K, Nagpal J, Laprell L, Schönberger M, Woodmansee DH, Rafiq A, Sumser MP, et al 2015. AzoCholine enables optical control of alpha 7 nicotinic acetylcholine receptors in neural networks. ACS Chem Neurosci. 6(5):701–707. doi:10.1021/acschemneuro.5b00030. 25741856

[jkaf086-B14] Del Marmol J, Yedlin MA, Ruta V. 2021. The structural basis of odorant recognition in insect olfactory receptors. Nature. 597(7874):126–131. doi:10.1038/s41586-021-03794-8. 34349260 PMC8410599

[jkaf086-B15] Durbin RM . 1987. Studies on the Development and Organisation of the Nervous System of Caenorhabditis elegans. Cambridge, United Kingdom: King's College Cambridge.

[jkaf086-B16] Eastman C, Horvitz HR, Jin Y. 1999. Coordinated transcriptional regulation of the unc-25 glutamic acid decarboxylase and the unc-47 GABA vesicular transporter by the Caenorhabditis elegans UNC-30 homeodomain protein. J Neurosci. 19(15):6225–6234. doi:10.1523/JNEUROSCI.19-15-06225.1999. 10414952 PMC6782798

[jkaf086-B17] Edelstein A, Amodaj N, Hoover K, Vale R, Stuurman N. 2010. Computer Control of Microscopes Using µManager. Hoboken, New Jersey, USA: John Wiley & Sons, Inc. 10.1002/0471142727.mb1420s92 PMC3065365 20890901

[jkaf086-B18] Edwards SL, Charlie NK, Milfort MC, Brown BS, Gravlin CN, Knecht JE, Miller KG. 2008. A novel molecular solution for ultraviolet light detection in Caenorhabditis elegans. PLoS Biol. 6(8):0060198. doi:10.1371/journal.pbio.0060198. PMC2494560 18687026

[jkaf086-B19] Evans R, O’Neill M, Pritzel A, Antropova N, Senior A, Green T, Žídek A, Bates R, Blackwell S, Yim J, et al 2022. Protein complex prediction with AlphaFold-Multimer. bioRxiv:2021.2010.2004.463034. 10.1101/2021.10.04.463034.

[jkaf086-B20] Garriga G, Desai C, Horvitz HR. 1993. Cell interactions control the direction of outgrowth, branching and fasciculation of the HSN axons of Caenorhabditis elegans. Development. 117(3):1071–1087. doi:10.1242/dev.117.3.1071. 8325236

[jkaf086-B21] Gendrel M, Atlas EG, Hobert O. 2016. A cellular and regulatory map of the GABAergic nervous system of C. elegans. Elife. 5:e17686. doi:10.7554/eLife.17686. 27740909 PMC5065314

[jkaf086-B22] Gong J, Yuan Y, Ward A, Kang L, Zhang B, Wu Z, Peng J, Feng Z, Liu J, Xu XZS. 2016. The C. elegans taste receptor homolog LITE-1 is a photoreceptor. Cell. 167(5):1252–1263.e1210. doi:10.1016/j.cell.2016.10.053. 27863243 PMC5388352

[jkaf086-B23] Goodale MA, Milner AD. 1992. Separate visual pathways for perception and action. Trends Neurosci. 15(1):20–25. doi:10.1016/0166-2236(92)90344-8. 1374953

[jkaf086-B24] Gray JM, Hill JJ, Bargmann CI. 2005. A circuit for navigation in Caenorhabditis elegans. Proc Natl Acad Sci U S A. 102(9):3184–3191. doi:10.1073/pnas.0409009101. 15689400 PMC546636

[jkaf086-B25] Hanson SM, Scholüke J, Liewald J, Sharma R, Ruse C, Engel M, Schüler C, Klaus A, Arghittu S, Baumbach F, et al 2023. Structure-function analysis suggests that the photoreceptor LITE-1 is a light-activated ion channel. Curr Biol. 33(16):3423–3435.e3425. doi:10.1016/j.cub.2023.07.008. 37527662

[jkaf086-B26] Heil K, Pearson D, Carell T. 2011. Chemical investigation of light induced DNA bipyrimidine damage and repair. Chem Soc Rev. 40(8):4271–4278. doi:10.1039/C000407N. 21076781

[jkaf086-B27] Klapoetke NC, Murata Y, Kim SS, Pulver SR, Birdsey-Benson A, Cho YK, Morimoto TK, Chuong AS, Carpenter EJ, Tian Z, et al 2014. Independent optical excitation of distinct neural populations. Nat Methods. 11(3):338–346. doi:10.1038/nmeth.2836. 24509633 PMC3943671

[jkaf086-B28] Land MF . 1999. Compound eye structure: matching eye to environment. In: Archer SN, Djamgoz MBA, Loew ER, Partridge JC, Vallerga S, editors. Adaptive Mechanisms in the Ecology of Vision. Dordrecht: Springer Netherlands. p. 51–71.

[jkaf086-B29] Liu J, Ward A, Gao J, Dong Y, Nishio N, Inada H, Kang L, Yu Y, Ma D, Xu T, et al 2010. C. elegans phototransduction requires a G protein-dependent cGMP pathway and a taste receptor homolog. Nat Neurosci. 13(6):715–722. doi:10.1038/nn.2540. 20436480 PMC2882063

[jkaf086-B30] Ludwig M, Leng G. 2006. Dendritic peptide release and peptide-dependent behaviours. Nat Rev Neurosci. 7(2):126–136. doi:10.1038/nrn1845. 16429122

[jkaf086-B31] McIntire SL, Jorgensen E, Kaplan J, Horvitz HR. 1993. The GABAergic nervous system of Caenorhabditis elegans. Nature. 364(6435):337–341. doi:10.1038/364337a0. 8332191

[jkaf086-B63] Mello CC, Kramer JM, Stinchcomb D, Ambros V. 1991. Efficient gene transfer in C. elegans: extrachromosomal maintenance and integration of transforming sequences. Embo J. 10:3959–3970. 1935914 10.1002/j.1460-2075.1991.tb04966.x PMC453137

[jkaf086-B32] Noma K, Jin Y. 2018. Rapid integration of multi-copy transgenes using optogenetic mutagenesis in Caenorhabditis elegans. G3 (Bethesda). 8(6):2091–2097. doi:10.1534/g3.118.200158. 29691291 PMC5982835

[jkaf086-B33] Oda K, Vierock J, Oishi S, Rodriguez-Rozada S, Taniguchi R, Yamashita K, Wiegert JS, Nishizawa T, Hegemann P, Nureki O. 2018. Crystal structure of the red light-activated channelrhodopsin Chrimson. Nat Commun. 9(1):3949. doi:10.1038/s41467-018-06421-9. 30258177 PMC6158191

[jkaf086-B34] Oranth A, Schultheis C, Tolstenkov O, Erbguth K, Nagpal J, Hain D, Brauner M, Wabnig S, Steuer Costa W, McWhirter RD, et al 2018. Food sensation modulates locomotion by dopamine and neuropeptide signaling in a distributed neuronal network. Neuron. 100(6):1414–1428.e1410. doi:10.1016/j.neuron.2018.10.024. 30392795

[jkaf086-B35] Oren-Suissa M, Bayer EA, Hobert O. 2016. Sex-specific pruning of neuronal synapses in Caenorhabditis elegans. Nature. 533(7602):206–211. doi:10.1038/nature17977. 27144354 PMC4865429

[jkaf086-B36] Pereira L, Kratsios P, Serrano-Saiz E, Sheftel H, Mayo AE, Hall DH, White JG, LeBoeuf B, Garcia LR, Alon U, et al 2015. A cellular and regulatory map of the cholinergic nervous system of C. elegans. Elife. 4:e12432. doi:10.7554/eLife.12432. 26705699 PMC4769160

[jkaf086-B37] Quintin S, Aspert T, Ye T, Charvin G. 2022. Distinct mechanisms underlie H2O2 sensing in C. elegans head and tail. PLoS One. 17(9):e0274226. doi:10.1371/journal.pone.0274226. 36173997 PMC9521893

[jkaf086-B38] Randi F, Sharma AK, Dvali S, Leifer AM. 2023. Neural signal propagation atlas of Caenorhabditis elegans. Nature. 623(7986):406–414. doi:10.1038/s41586-023-06683-4. 37914938 PMC10632145

[jkaf086-B39] Ren XC, Kim S, Fox E, Hedgecock EM, Wadsworth WG. 1999. Role of netrin UNC-6 in patterning the longitudinal nerves of Caenorhabditis elegans. J Neurobiol. 39(1):107–118. doi:10.1002/(SICI)1097-4695(199904)39:1<107::AID-NEU9>3.0.CO;2-7. 10213457

[jkaf086-B40] Riddle DL, Blumenthal T, Meyer BJ, Priess JR. 1997. Section IV Hierachies oF Guidance Cues in C. elegans II. 2nd ed. NY: Cold Spring Harbor Laboratory Press, Cold Spring Harbor.

[jkaf086-B41] Ripoll-Sánchez L, Watteyne J, Sun H, Fernandez R, Taylor SR, Weinreb A, Bentley BL, Hammarlund M, Miller DM, Hobert O, et al 2023. The neuropeptidergic connectome of C. elegans. Neuron. 111(22):3570–3589.e3575. doi:10.1016/j.neuron.2023.09.043. 37935195 PMC7615469

[jkaf086-B42] Rupnik M, Kreft M, Sikdar SK, Grilc S, Romih R, Zupancic G, Martin TF, Zorec R. 2000. Rapid regulated dense-core vesicle exocytosis requires the CAPS protein. Proc Natl Acad Sci U S A. 97(10):5627–5632. doi:10.1073/pnas.090359097. 10792045 PMC25879

[jkaf086-B43] Saina M, Busengdal H, Sinigaglia C, Petrone L, Oliveri P, Rentzsch F, Benton R. 2015. A cnidarian homologue of an insect gustatory receptor functions in developmental body patterning. Nat Commun. 6(1):6243. doi:10.1038/ncomms7243. 25692633 PMC4374167

[jkaf086-B44] Setty H, Salzberg Y, Karimi S, Berent-Barzel E, Krieg M, Oren-Suissa M. 2022. Sexually dimorphic architecture and function of a mechanosensory circuit in C. elegans. Nat Commun. 13(1):6825. doi:10.1038/s41467-022-34661-3. 36369281 PMC9652301

[jkaf086-B45] Sieburth D, Madison JM, Kaplan JM. 2007. PKC-1 regulates secretion of neuropeptides. Nat Neurosci. 10(1):49–57. doi:10.1038/nn1810. 17128266

[jkaf086-B46] Smith SJ, Sümbül U, Graybuck LT, Collman F, Seshamani S, Gala R, Gliko O, Elabbady L, Miller JA, Bakken TE, et al 2019. Single-cell transcriptomic evidence for dense intracortical neuropeptide networks. Elife. 8:e47889. doi:10.7554/eLife.47889. 31710287 PMC6881117

[jkaf086-B47] Speese S, Petrie M, Schuske K, Ailion M, Ann K, Iwasaki K, Jorgensen EM, Martin TFJ. 2007. UNC-31 (CAPS) is required for dense-core vesicle but not synaptic vesicle exocytosis in Caenorhabditis elegans. J Neurosci. 27(23):6150–6162. doi:10.1523/JNEUROSCI.1466-07.2007. 17553987 PMC6672138

[jkaf086-B48] Steuer Costa W, Yu SC, Liewald JF, Gottschalk A. 2017. Fast cAMP modulation of neurotransmission via neuropeptide signals and vesicle loading. Curr Biol. 27(4):495–507. doi:10.1016/j.cub.2016.12.055. 28162892

[jkaf086-B49] Stirman JN, Crane MM, Husson SJ, Gottschalk A, Lu H. 2012. A multispectral optical illumination system with precise spatiotemporal control for the manipulation of optogenetic reagents. Nat Protoc. 7(2):207–220. doi:10.1038/nprot.2011.433. 22240583 PMC8102134

[jkaf086-B65] Swierczek NA, Giles AC, Rankin CH, Kerr RA. 2011. High-throughput behavioral analysis in C. elegans. Nat Methods. 8:592–598. 21642964 10.1038/nmeth.1625 PMC3128206

[jkaf086-B50] Taylor JS . 1994. Unraveling the molecular pathway from sunlight to skin cancer. Acc Chem Res. 27(3):76–82. doi:10.1021/ar00039a003.

[jkaf086-B51] Taylor SR, Santpere G, Weinreb A, Barrett A, Reilly MB, Xu C, Varol E, Oikonomou P, Glenwinkel L, McWhirter R, et al 2021. Molecular topography of an entire nervous system. Cell. 184(16):4329–4347.e4323. doi:10.1016/j.cell.2021.06.023. 34237253 PMC8710130

[jkaf086-B52] Ungerleider LG, Mishkin M. 1982. Two cortical visual systems. Analysis of visual behavior. Cambridge, Massachusetts, USA: MIT Press. p. 549.

[jkaf086-B53] van den Pol AN . 2012. Neuropeptide transmission in brain circuits. Neuron. 76(1):98–115. doi:10.1016/j.neuron.2012.09.014. 23040809 PMC3918222

[jkaf086-B54] Vettkötter D, Schneider M, Goulden BD, Dill H, Liewald J, Zeiler S, Guldan J, Ateş YA, Watanabe S, Gottschalk A. 2022. Rapid and reversible optogenetic silencing of synaptic transmission by clustering of synaptic vesicles. Nat Commun. 13(1):7827. doi:10.1038/s41467-022-35324-z. 36535932 PMC9763335

[jkaf086-B55] Wang C, Vidal B, Sural S, Loer C, Aguilar GR, Merritt DM, Toker IA, Vogt MC, Cros CC, Hobert O. 2024. A neurotransmitter atlas of C. elegans males and hermaphrodites. eLife. 13:RP95402. doi:10.7554/eLife.95402. 39422452 PMC11488851

[jkaf086-B56] Ward A, Liu J, Feng Z, Xu XZ. 2008. Light-sensitive neurons and channels mediate phototaxis in C. elegans. Nat Neurosci. 11(8):916–922. doi:10.1038/nn.2155. 18604203 PMC2652401

[jkaf086-B57] White JG, Southgate E, Thomson JN, Brenner S. 1986. The structure of the nervous system of the nematode Caenorhabditis elegans. Philos Trans R Soc Lond B Biol Sci. 314(1165):1–340. doi:10.1098/rstb.1986.0056. 22462104

[jkaf086-B58] Witvliet D, Mulcahy B, Mitchell JK, Meirovitch Y, Berger DR, Wu Y, Liu Y, Koh WX, Parvathala R, Holmyard D, et al 2021. Connectomes across development reveal principles of brain maturation. Nature. 596(7871):257–261. doi:10.1038/s41586-021-03778-8. 34349261 PMC8756380

[jkaf086-B59] Xiang Y, Yuan Q, Vogt N, Looger LL, Jan LY, Jan YN. 2010. Light-avoidance-mediating photoreceptors tile the Drosophila larval body wall. Nature. 468(7326):921–926. doi:10.1038/nature09576. 21068723 PMC3026603

[jkaf086-B60] Yu SC, Liewald JF, Shao J, Steuer Costa W, Gottschalk A. 2021. Synapsin is required for dense core vesicle capture and cAMP-dependent neuropeptide release. J Neurosci. 41(19):4187–4201. doi:10.1523/JNEUROSCI.2631-20.2021. 33820857 PMC8143207

