# Supplementary material for: Neurons and molecules involved in noxious light sensation in Caenorhabditis elegans
Source: G3 (Bethesda). 2025 Apr 16;15(6):jkaf086. doi: 10.1093/g3journal/jkaf086 (PMC12135013; doi:10.1093/g3journal/jkaf086)
Supplement: jkaf086_Supplementary_Data [file jkaf086_supplementary_data.zip › Supplementary_File_S1_G3-2025-405808.pdf]

# Neurons and molecules involved in noxious light sensation in *Caenorhabditis elegans*

Eva Dunkel[1,2], Ichiro Aoki[1,2], Amelie Bergs[1,2], Alexander Gottschalk[1,2,*]

1 Buchmann Institute of Molecular Life Sciences, Goethe University, Max-von-Laue-Strasse 15, D-60438 Frankfurt, Germany

2 Institute for Biophysical Chemistry, Goethe University, Max-von-Laue-Strasse 9, D-60438 Frankfurt, Germany

* to whom correspondence should be addressed: a.gottschalk@em.uni-frankfurt.de

## Supplemental Figures

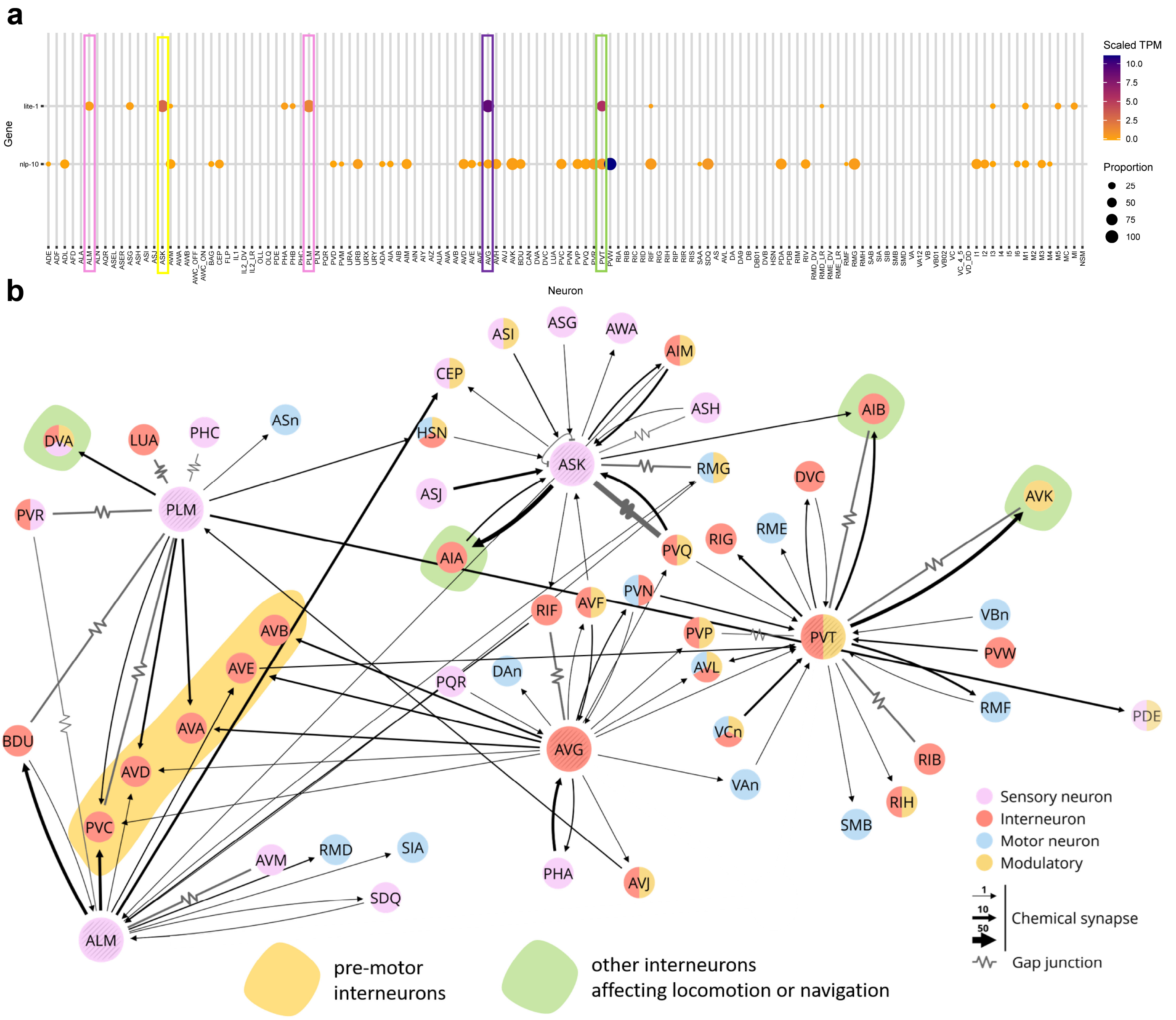


**Figure S1: Level of gene expression of LITE-1 and NLP-10. (A)** Visualization of gene expression level of LITE-1 and NLP-10. Highlighted are the neurons investigated in the study: AVG (purple), PVT (green), ASK (yellow), as well as ALM and PLM (pink). Data adopted and amended from CenGEN database (Taylor et al., 2021; https://cengen.shinyapps.io/CengenApp/). **(B)** Networks of neurons innervated by the AVG, PVT, ASK, ALM, and PLM neurons, extracted and modified from www.nemanode.org (Witvliet et al., 2021).

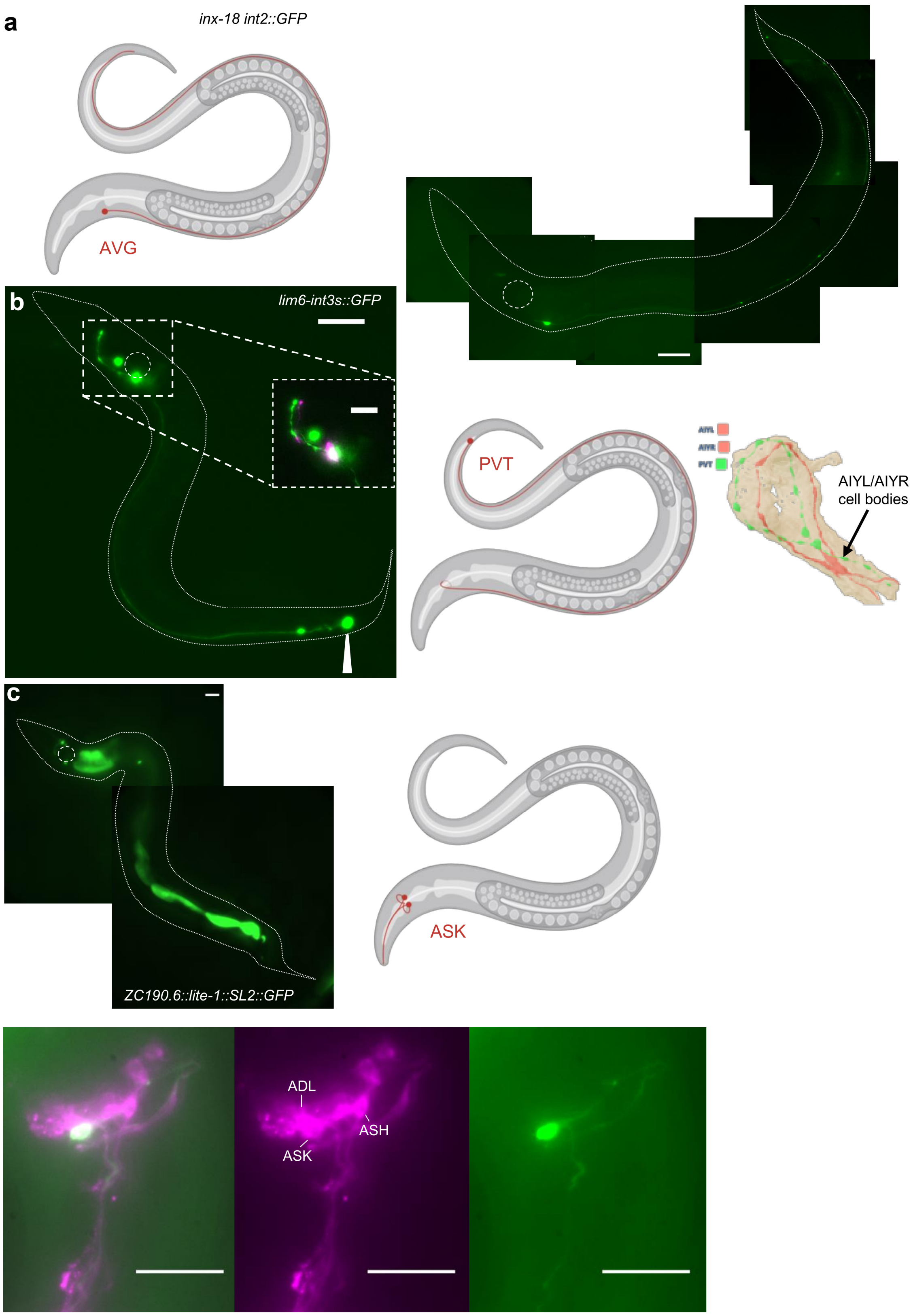


**Figure S2: Expression pattern of promoters driving GFP in AVG, PVT and ASK neurons. (A)** Schematic representation of AVG in the organism, indicated in red (Biorender license GZ27OPINLR). Representative images of GFP fluorescence expressed in AVG using intron 2 sequences from the *inx-18* gene. The outline of the animal has been traced with a dashed line; the pharyngeal terminal bulb is indicated by a circle. Scale bar: 50 µm. **(B)** Left: Representative images of GFP fluorescence signal expressed in PVT using intron 3 sequences from the *lim-6* gene. The outline of the animal has been traced with a dashed line; the terminal bulb is indicated by a circle. Scale bar: 50 µm. Close up of the head region of the animal reveals partial co-localization with pAIY::mScarlet, co-injected as marker. Scale bar: 25 µm. Middle: Schematic representation of AIY(L/R) cell

bodies and processes (red) and PVT process with large varicosities (green) in the nerve ring, from www.nemanode.org. Right: Schematic representation of PVT in the organism, indicated in red (Biorender license CP27OPIWPX). **(C)** Top left: Representative images of LITE-1::SL2::GFP fluorescence signal expressed in ASK from the promoter of the *ZC190.6* gene. The outline of the animal has been traced with a dashed line; the terminal bulb is indicated by a circle. Scale bar: 50 µm. Note that the SL2 spliced leader sequence drives GFP expression also in the gut. Top right: Schematic representation of ASK in the organism, indicated in red (Biorender license OW27OPJ1QN). Bottom: Representative images of DiI staining to verify expression in ASK and other ciliated neurons, as indicated (magenta, middle). Green channel (right) shows pASK::GFP expression, and overlay is shown on the (left). Anterior is down, ventral right. Worm pictograms were generated using Biorender and modified.

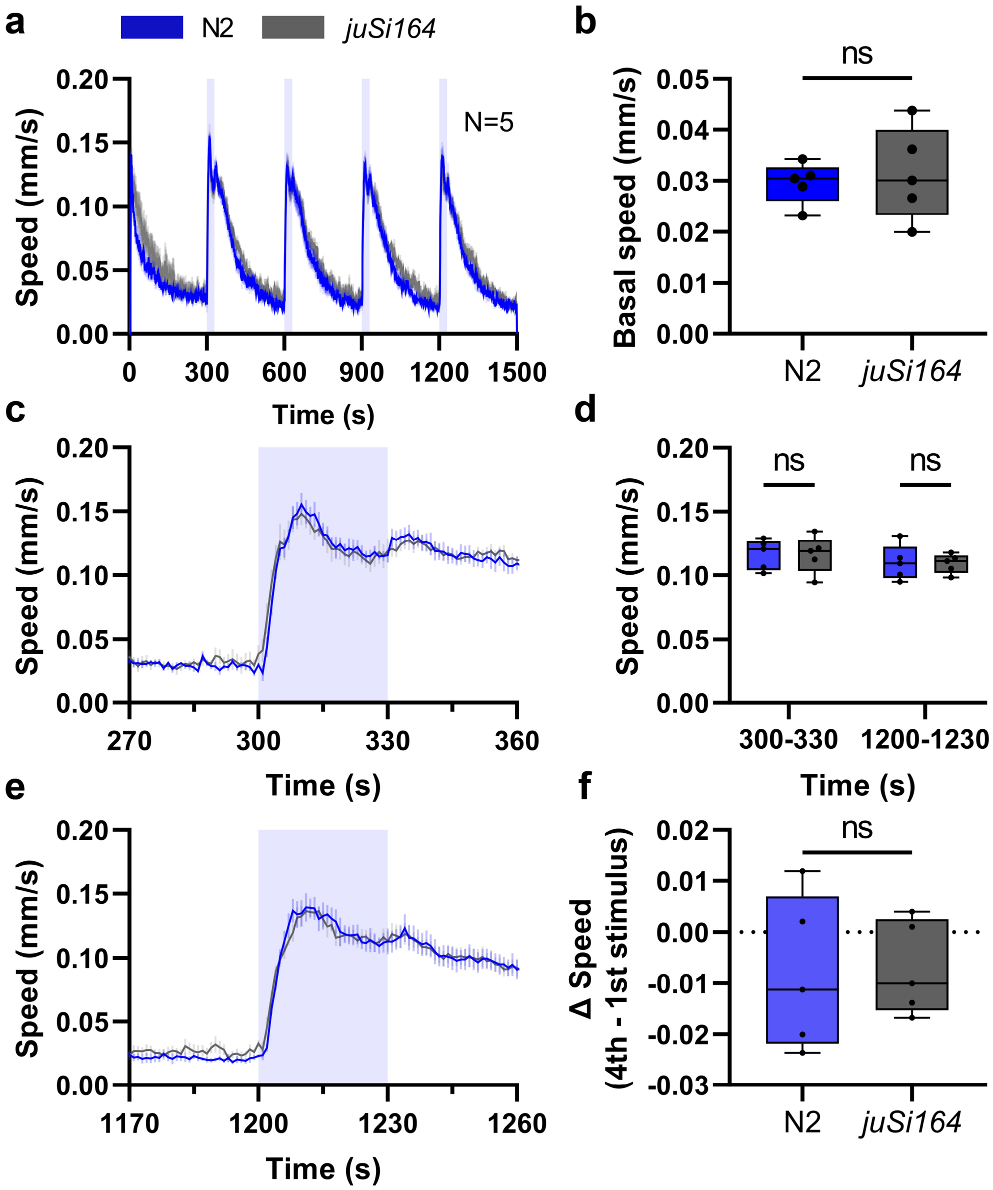


**Figure S3:** ***juSi164* animals mimic locomotive behavior of wild type N2 animals.** (**A**) Mean crawling speed (± SEM) of animals during repetitive blue light stimulation at 300-330, 600-630, 900-930 and 1200-1230 s, as indicated by blue bars. Tested strains were N2 wild type and *juSi164.* N=5 with 22-35 animals each. (**B**) Mean basal speed before first illumination (270-299 s). Median with 25/75 quartiles, whiskers indicate minimum and maximum values. Unpaired t-test (ns = non-significant). (**C**) Close-up of locomotion speed 30 s before, during and after the first illumination period. (**D**) Mean speed during first and fourth illumination. Two-way ANOVA with Bonferroni as post-hoc analysis (ns = not significant). (**E**) Close-up of locomotion speed 30 s before, during and after the fourth illumination period. (**F**) Difference of speed level upon first vs. fourth illumination, mean speed during the fourth illumination was subtracted from mean speed during the first illumination. Median with 25/75 quartiles, whiskers indicate minimum and maximum values. Unpaired, two-tailed t-test (ns = not significant).

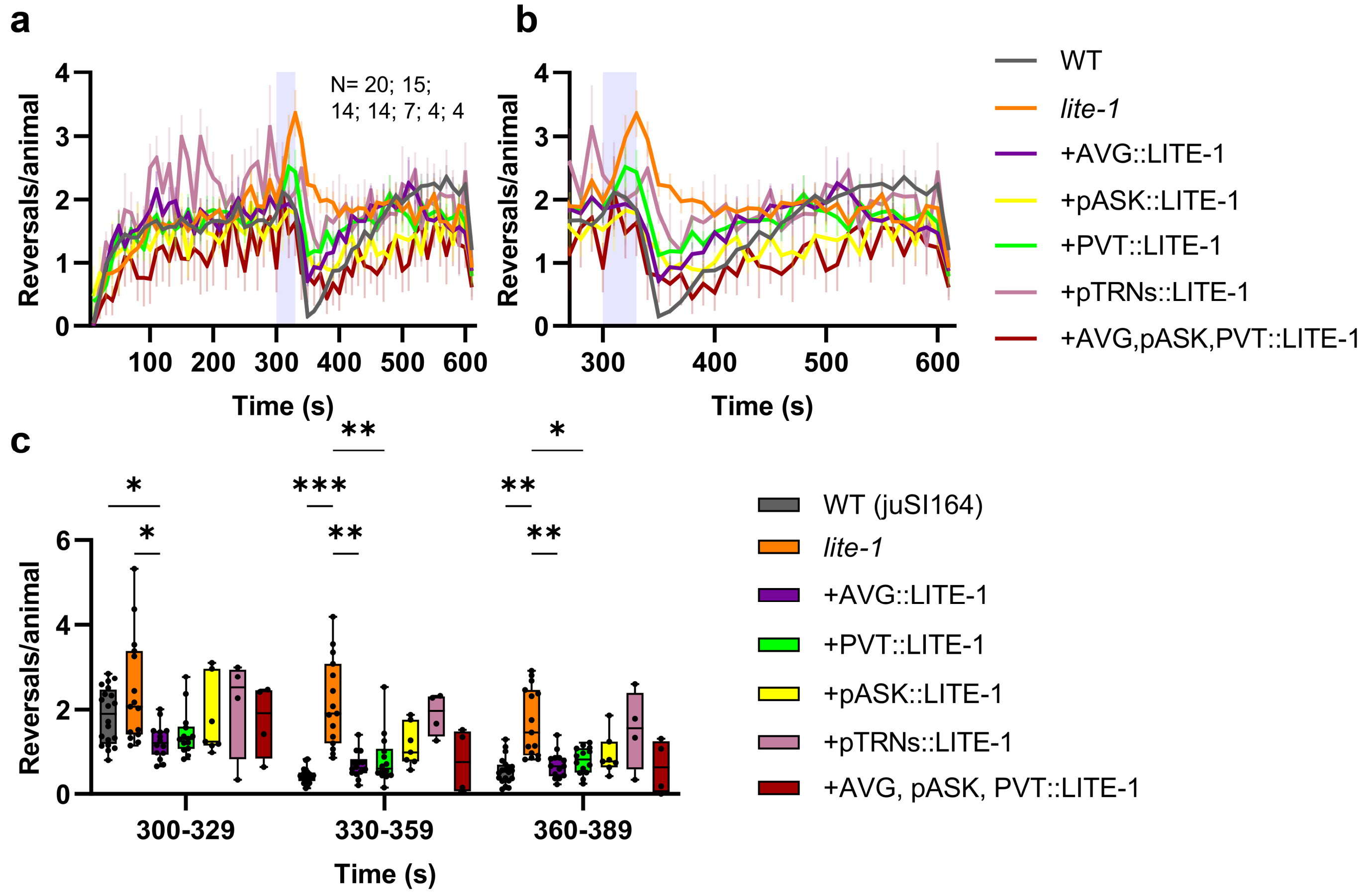


**Figure S4: Reversal rate of LITE-1 expressing animals before, during and after illumination.** (**A**) Datasets as in **Fig. 1** were analyzed for (time involved in) reversal locomotion per animal per 10 sec. Tested strains were wild type, *lite-1* and animals expressing LITE-1 in either AVG, PVT, ASK or TRNs, as well as animals expressing LITE-1 in AVG, ASK and PVT simultaneously. N=20, 15, 14, 14, 7, 4, and 4 experiments with 20-32 animals for each measurement, respectively. (**B**) Close up of reversal data per animal between 270 and 600 s. (**C**) Reversals/animals during (300-329 s) and after (330-359 s and 360-389 s). Median with 25/75 quartiles, whiskers indicate minimum and maximum values. Two-way ANOVA with Tukey test ($p < 0.05$ = *; $p < 0.01$ = **; $p < 0.001$ = ***).

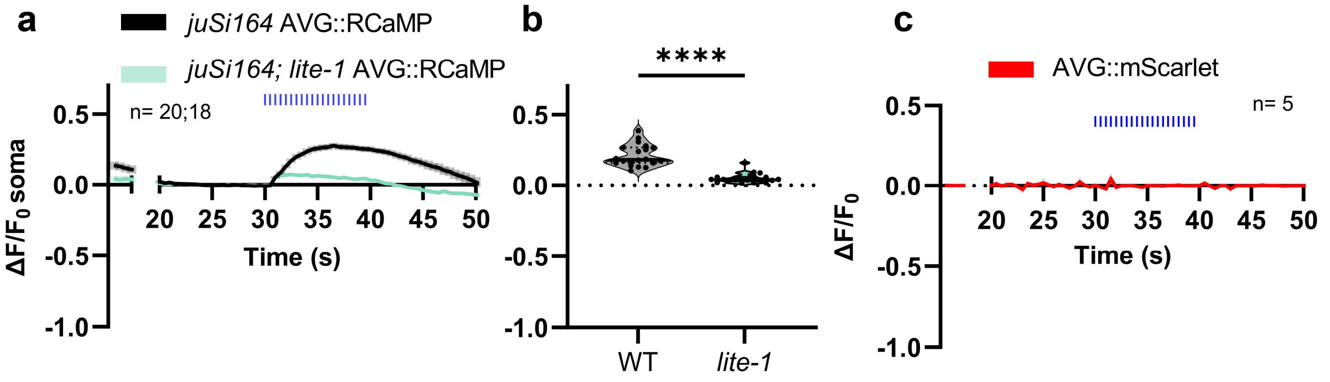


**Figure S5: $Ca^{2+}$ influx in AVG soma upon blue light illumination.** (**A**) Mean (±SEM) change of fluorescence intensity of RCaMP in the AVG soma before (20-29 s), during (30-39 s) and after (40-50 s) application of blue light pulses (indicated by blue bars). n=20 animals for wild type and n=18 for *lite-1*. $F_0$ was calculated during seconds 20-29. (**B**) Mean RCaMP signal intensity during blue light application of individual animals. Median (thick line) and 25/75 quartiles (dotted or thin lines). Unpaired t-test ($p < 0.0001$ = ****). (**C**) Mean (±SEM) change of fluorescence intensity of ($Ca^{2+}$-insensitive) mScarlet, expressed in AVG before (20-29 s), during (30-39 s) and after (40-50 s) application of blue light pulses (indicated by blue bars. $F_0$ was calculated for sec 20 – 29. n= 5.

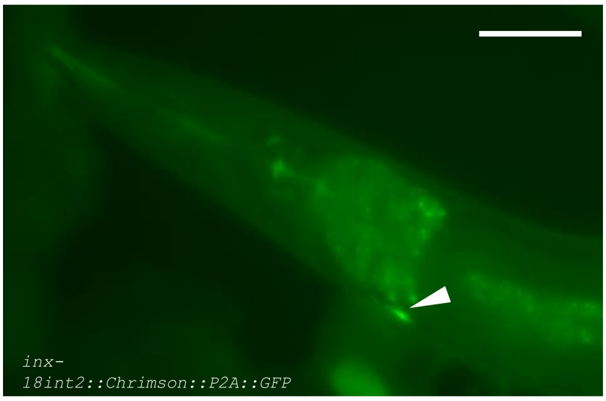


**Figure S6**: **Verification of Chrimson expression in AVG.** Representative image of Chrimson::GFP fluorescence signal expressed under the AVG promoter using sequences from intron 2 of the *inx-18* gene. Scale bar indicates 50 µm.

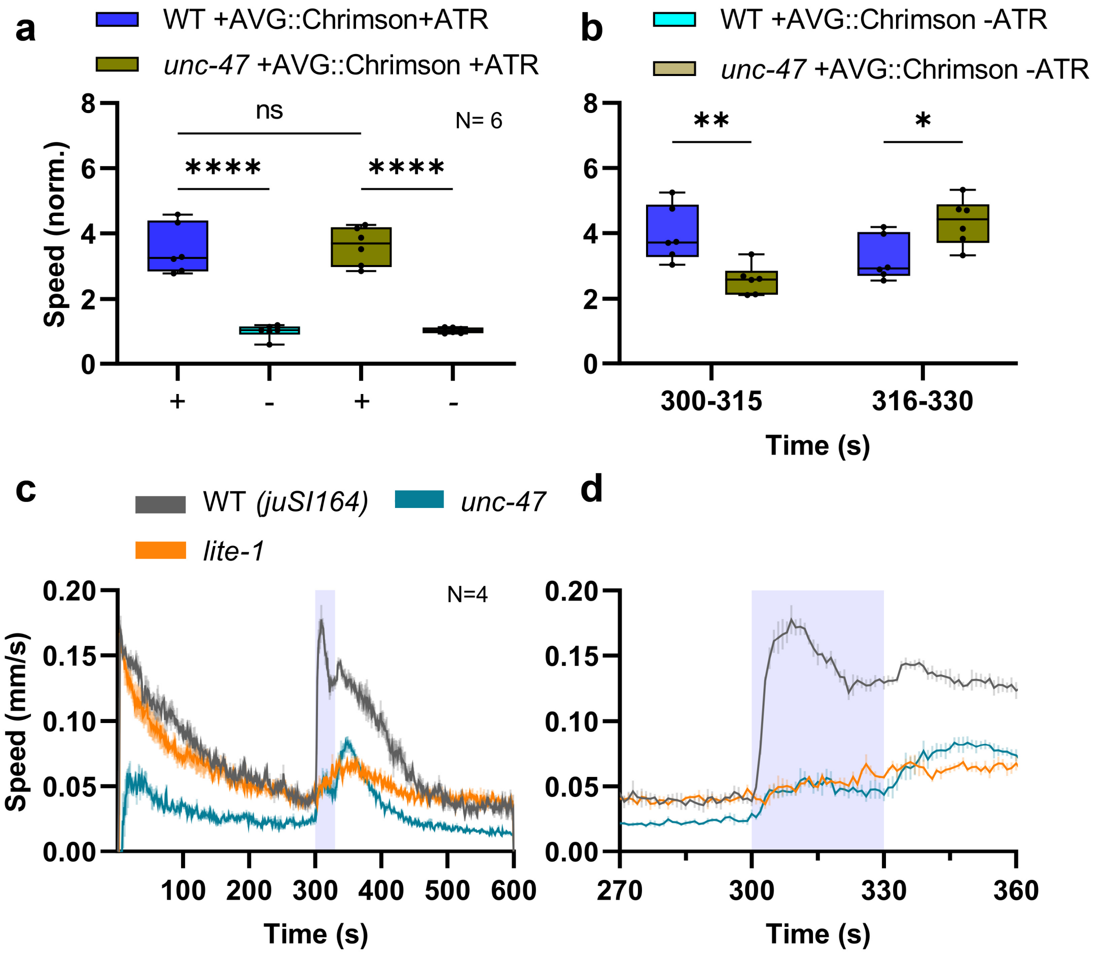


**Figure S7**: ***unc-47* mutants show weaker immediate speed increase upon Chrimson activation in AVG and to noxious blue light illumination than wild type.** (**A**) Mean crawling speed of animals during red light illumination. Wild type animals and *unc-47(e307)* mutants expressing Chrimson in AVG, that were either exposed to ATR, or not (indicated by + and – respectively). Two-way ANOVA with Tukey post-hoc analysis (ns = not significant; $p < 0.0001$ = ****). N=6 with 26 – 31 animals each. (**B**) Mean crawling speed during the first and last 15 s of illumination. Median with 25/75 quartiles, whiskers indicate minimum and maximum values. Two-way ANOVA with Bonferroni's test was performed ($p < 0.05$ = *; $p < 0.01$ = **). (**C**) Mean crawling speed (± SEM) of wild type, *lite-1* and *unc-47* animals before (0-299 s), during (300-330 s) and after (331-600 s) blue light illumination. Illumination is indicated by the blue shade. N=4 experiments with 22-32 animals each. (**D**) Close-up of locomotion speed 30 s before, during and after illumination.

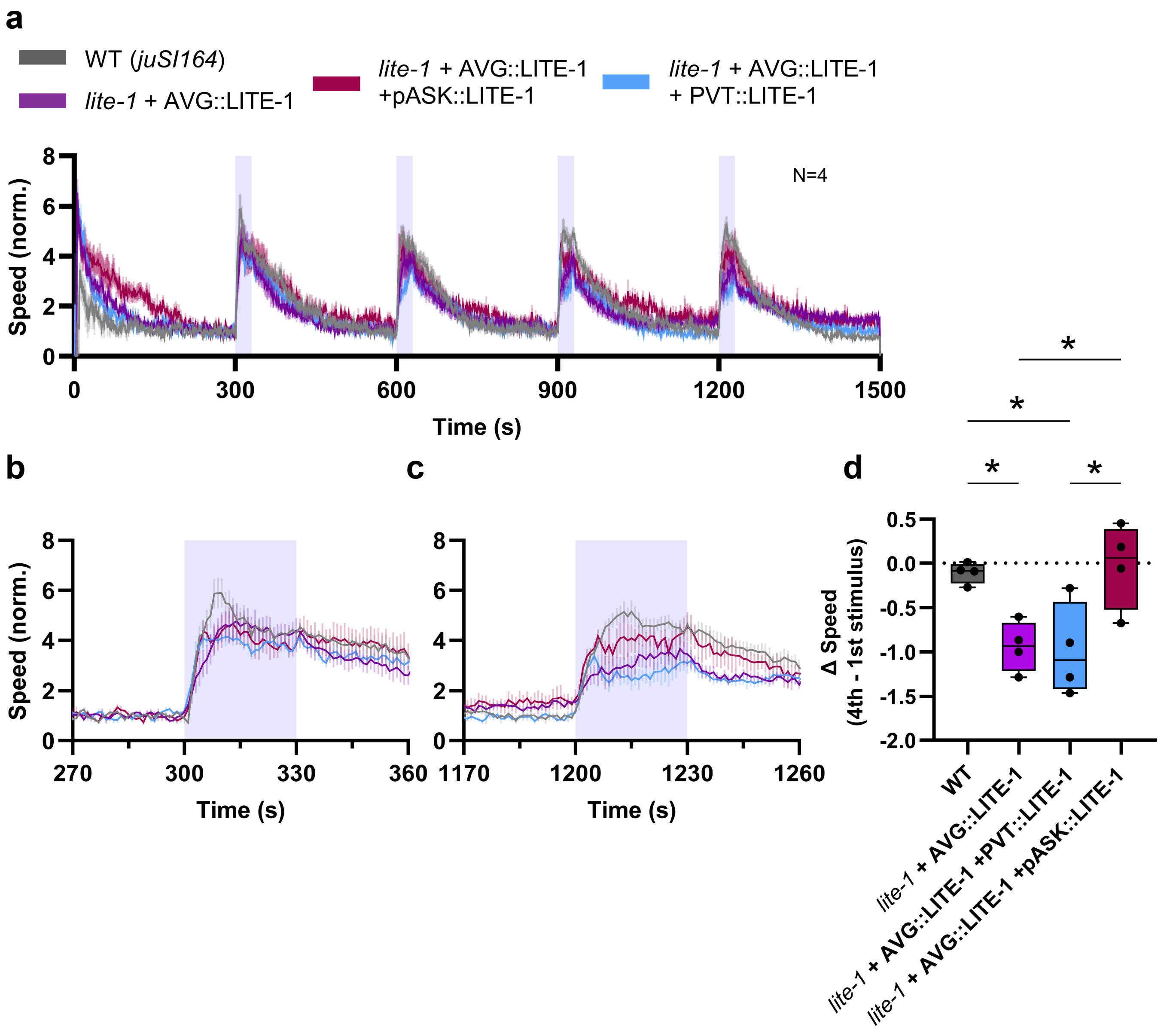


**Figure S8: Loss of sensitivity to repetitive stimulation was rescued by expressing LITE-1 in ASK.** (**A**) Mean crawling speed (± SEM) of the animals during repetitive blue light stimulation at 300-330, 600-630, 900-930 and 1200-1230 s, as indicated by blue shades. Crawling speed was normalized to the mean crawling speed from 270 to 299 s. Tested strains were wild type, as well as *lite-1* mutants either expressing solely LITE-1 in AVG, or additionally in PVT or ASK. N=4 with 23-31 animals each. (**B, C**) Close-ups of locomotion speed 30 s before, during and after the first and the fourth illumination period, respectively. (**D**) Differences of mean speeds of fourth illumination, deduced by speed levels at first illumination. Median with 25/75 quartiles, whiskers indicate minimum and maximum values. One-way ANOVA with Tukey test ($p < 0.05$ = *; $p < 0.01$ = **; $p < 0.001$ = ***; $p < 0.0001$ = ****).

## Further Supplemental Material

**Table S1: Expression levels of mRNAs of *nlp-10* and *lite-1*.** (**A**) Cell types ranked by expression of *nlp-10* mRNA. (**B**) Cell types ranked by expression of *lite-1* mRNA. Red highlighted cells were studied in this work. Data extracted from CenGEN database (Taylor et al., 2021; https://cengen.shinyapps.io/CengenApp/).

**a**

| **#** | **Cell type** | **Expression level of *nlp-10* mRNA** |
|---|---|---|
| 1 | PVW | 73721.981 |
| 2 | SDQ | 4587.179 |
| 3 | **PVT** | 4284.468 |
| 4 | PVR | 3281.666 |
| 5 | RIF | 3115.366 |
| 6 | RMG | 2711.265 |
| 7 | URB | 2425.919 |
| 8 | AVK | 2125.116 |
| 9 | PDA | 2006.543 |
| 10 | AVD | 1681.321 |
| 11 | AVH | 1577.412 |
| 12 | PVC | 1504.545 |
| 13 | I1 | 1455.747 |
| 14 | I2 | 1437.14 |
| 15 | BDU | 1396.143 |
| 16 | RIV | 1361.985 |
| 17 | PVP | 1161.3 |
| 18 | AIM | 1133.435 |
| 19 | CEP | 1132.049 |
| 20 | M3 | 968.59 |
| 21 | PVQ | 943.981 |
| 22 | **AVG** | 859.37 |
| 23 | AVE | 671.065 |
| 24 | I6 | 616.909 |
| 25 | AVM | 583.973 |
| 26 | PVM | 445.842 |
| 27 | ADL | 432.909 |
| 28 | AIA | 320.761 |
| 29 | ADA | 304.149 |
| 30 | PVD | 281.316 |
| 31 | BAG | 207.878 |
| 32 | I3 | 197.844 |
| 33 | M1 | 134.116 |
| 34 | ADE | 125.593 |
| 35 | AVF | 88.533 |
| 36 | SAA | 88.306 |
| 37 | RMF | 51.03 |
| 38 | M4 | 49.427 |

**b**

| **#** | **Cell type** | **Expression level of *lite-1* mRNA** |
|---|---|---|
| 1 | **AVG** | 7827.058 |
| 2 | **PVT** | 4890.871 |
| 3 | **ASK** | 2711.229 |
| 4 | **PLM** | 1399.659 |
| 5 | PHA | 333.203 |
| 6 | **ALM** | 315.035 |
| 7 | RIF | 270.771 |
| 8 | MI | 266.455 |
| 9 | PHB | 262.341 |
| 10 | ASG | 231.55 |
| 11 | M5 | 204.111 |
| 12 | M1 | 94.329 |
| 13 | RMD_LR | 61.803 |
| 14 | I3 | 41.664 |
| 15 | AVM | 28.588 |

**Data File S1:** Data from on-line measurements of the multiworm tracker are summarized, sorted for each figure of the paper, as well as imaging data and statistical analysis.

**Supplemental Code:** µManager Beanshell scripts for selective illumination using a video projector:

Supplemental code 1 “Projector_calibration.bsh”

Supplemental code 2 “Pulse_protocol_patterned_stimulation.bsh”

## Supplemental Videos

**Video S1: Locomotive behavior of wild type (*juSi164*) animals before, during and after blue light illumination.** Close-up of the tested plate before (0-9 s), during (10-39 s) and after (40-50 s). Wild type (*juSi164*) animals were transferred onto the seeded (OP-50) plates 16 h prior to experiment. Recording started after 290 s to ensure a similar basal speed as in experiments. Blue point indicates the illumination period.

**Video S2: Locomotive behavior of *lite-1(ce314)* animals before, during and after blue light illumination.** Close-up of the tested plate before (0-9 s), during (10-39 s) and after (40-50 s). *lite-1(ce314)* animals were transferred onto the seeded (OP-50) plates 16 h prior to experiment. Recording started after 290 s to ensure a similar basal speed as in experiments. Blue point indicates the illumination period.

**Video S3: Locomotive behavior of wild type (*juSi164*) animals expressing Chrimson in AVG before, during and after red light illumination.** Close-up of the tested plate before (0-9 s), during (10-39 s) and after (40-50 s). Wild type (*juSi164*) animals were transferred onto the seeded (OP-50+ATR) plates 16 h prior to experiment. Recording started after 290 s to ensure a similar basal speed as in experiments. Red point indicates the illumination period.

**Video S4: Locomotive behavior of *unc-47(e307)* animals expressing Chrimson in AVG before, during and after red light illumination.** Close-up of the tested plate before (0-9 s), during (10-39 s) and after (40-50 s). *unc-47(e307)* animals were transferred onto the seeded (OP-50+ATR) plates 16 h prior to experiment. Recording started after 290 s to ensure a similar basal speed as in experiments. Red point indicates the illumination period.
